# Aeciospore ejection in the rust pathogen *Puccinia graminis* is driven by moisture ingress

**DOI:** 10.1038/s42003-021-02747-1

**Published:** 2021-10-22

**Authors:** Vanessa Bueno-Sancho, Elizabeth S. Orton, Morgan Gerrity, Clare M. Lewis, Phoebe Davey, Kim C. Findlay, Elaine Barclay, Phil Robinson, Richard J. Morris, Mark Blyth, Diane G. O. Saunders

**Affiliations:** 1 John Innes Centre, Norwich Research Park, Norwich, NR4 7UH UK; 2 University of East Anglia, Norwich Research Park, Norwich, NR4 7TJ UK

**Keywords:** Pathogens, Fungal biology

## Abstract

Fungi have evolved an array of spore discharge and dispersal processes. Here, we developed a theoretical model that explains the ejection mechanics of aeciospore liberation in the stem rust pathogen *Puccinia graminis*. Aeciospores are released from cluster cups formed on its *Berberis* host, spreading early-season inoculum into neighboring small-grain crops. Our model illustrates that during dew or rainfall, changes in aeciospore turgidity exerts substantial force on neighboring aeciospores in cluster cups whilst gaps between spores become perfused with water. This perfusion coats aeciospores with a lubrication film that facilitates expulsion, with single aeciospores reaching speeds of 0.053 to 0.754 m**·**s^−1^. We also used aeciospore source strength estimates to simulate the aeciospore dispersal gradient and incorporated this into a publicly available web interface. This aids farmers and legislators to assess current local risk of dispersal and facilitates development of sophisticated epidemiological models to potentially curtail stem rust epidemics originating on *Berberis*.

## Abstract (web summary)

Bueno-Sancho et al. use high-speed videography and mathematical modelling to examine aeciospore dispersal mechanics of the stem rust fungus *Puccinia graminis*. Their model shows that aeciospore ejection is driven by peridium rupture and moisture ingress, with dispersal projections encapsulated in a web interface to help assess risk of disease spread to nearby cereal crops.

## Introduction

Fungal diseases pose a major threat to global food security^1^, and the fungi responsible for these diseases utilize an array of spore discharge and dispersal mechanisms^2^. Mechanistic knowledge of spore ejection (“liberation”) processes can elucidate the key environmental criteria—and the timing—for spore release^3^. Epidemiological models can then be used to infer disease transmission leading to predictions regarding the distance of spore travel^4^, likely timing of subsequent infection^3^, and development of disease foci across fields or even continents^5^. Once spores of plant-associated fungi are liberated, they are dispersed via water, soil, vectors, or air (depending on their location in the plant), and the dispersal mode strongly influences the subsequent dispersal gradient^6^. When spores are liberated from leaves into the air (foliar aerial liberation), the first step involves the release of spores from parental tissue, a process that can be active or passive. The best-known example of active ejection is the surface-tension catapult mechanism for the discharge of ballistospores by basidiomycete fungi. In this case, a fluid-filled “Buller’s drop” forms at the spore’s hilar appendix via condensation of water on the spore surface, and its fusion with a second expanding adaxial drop causes a shift in the center of spore mass, resulting in spore launch^7^. However, for many spore types, the mechanics of active release have remained elusive despite the importance of this information for fungal epidemiology.

Rust fungi (order Pucciniales) are the largest group of plant-parasitic fungi and include many species of agricultural importance, such as the notorious stem rust fungus, *Puccinia graminis* (*Pg*), which has caused dramatic crop failures throughout history^8^. Like other rust fungi, *Pg* is heteroecious and undergoes asexual reproduction on small-grain crops and grasses through cycles of urediniospore production^9^. Urediniospores are passively released and can be liberated through raindrop impact, which creates an air vortex ring that increases the height and distance of subsequent long-distance dry dispersal^10^. At the end of the growing season for the grain/grass host, *Pg* switches to produce hardy overwintering teliospores on plant debris. The teliospores germinate in the spring to form basidiospores that infect the unrelated shrub *Berberis*. Infection of *Berberis* gives rise to flask‐shaped pycnia on the upper surface of its leaves, and then sexual recombination produces genetically diverse *Pg* aeciospores that are tightly packed in cluster cups (“aecia”) on the underside of the *Berberis* leaves^9^. In temperate zones, aeciospores act as the only source of early-season wind-borne inoculum to begin the disease cycle again on gramineous hosts in the spring^11^. Over the past century, the crucial role of *Berberis* species in the wheat stem rust lifecycle led to extensive legislation and exclusion campaigns to eradicate the highly susceptible species *B. vulgaris* from wheat-growing areas in many countries across Europe and North America^12^. These eradication efforts were extremely successful in reducing early-season inoculum and the number of new races by diminishing sexual recombination, as demonstrated in the US where the number of *P. graminis* f. sp. *tritici* races per year declined from 17 to 8 after *B. vulgaris* eradication^13^.

It has long been recognized that local dispersal of *Pg* aeciospores makes the practice of planting susceptible *Berberis* species near grain/grass host plants severely risky to those crops (e.g.^14,15^). However, long-distance dispersal can also occur, with small numbers of aeciospores becoming trapped in wind currents and traveling up to 500 km, and their thick outer walls facilitate long-distance survival^16,17^. Aeciospore liberation for *Pg* is also known to occur under high relative humidity, with aeciospore expansion and subsequent changes in turgidity suggested to be likely factors underpinning forceable active expulsion^18,19^. However, the precise mechanics of aeciospore discharge are unknown.

Here, we carried out an extensive evaluation of the mechanics of aeciospore release for *Pg* and developed a theoretical model to describe this process. We successfully validated the model using high-speed videography. Based on these results, we conclude that hydration causes gaps between spores (i.e., interspore gaps) to become perfused with water, creating a thin fluid film lubrication between aeciospores, reducing their dry state friction and propelling single aeciospores to escape the leaf boundary layer of still air at speeds ranging from 0.053 to 0.754 m s^−1^, in a similar manner to a squeeze catapult. Furthermore, we evaluated the number of aeciospores produced per *B. vulgaris* bush, employed a Gaussian Plume model to simulate the potential dispersal gradient, and incorporated this model into a point-and-click web interface available to the public. Our results will help guide modern-day legislation around *B. vulgaris* planting and promote the development of more accurate epidemiological risk models to protect cereal crops from early-season inoculum, potentially reducing the severity of future *Pg* epidemics.

## Results

### Free water is required to facilitate aeciospore expansion

**Fig. 1:**
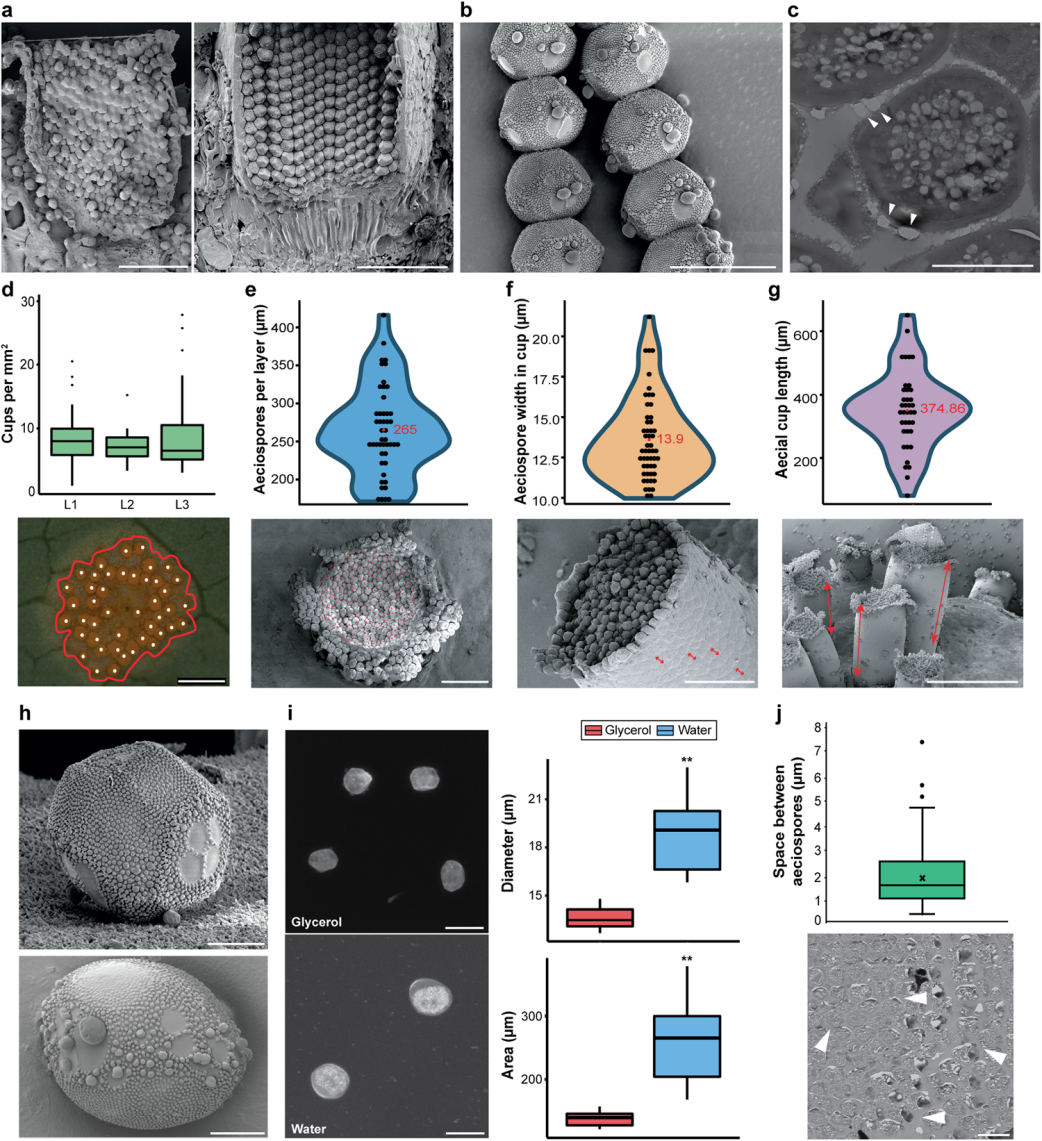
Polyhedral Pg aeciospores are tightly packed in aecial cups and expand, rounding-off when immersed in water. **a**, **b** Scanning electron micrographs of angular *Pg* aeciospores, tightly packed inside aecial cups, were frequently identified in chains once expelled onto the leaf surface. Aeciospores were also coated in verrucae that formed characteristic plugs on the aeciospore surface. Scale bars: 100 µm (aecial cups) and 20 µm (aeciospore chain). **c** Transmission electron micrograph of *Pg* aeciospores. White arrowheads indicate verrucae plugs. Scale bar: 5 µm. **d** Analysis of 129 *Pg* aecia from three independent locations (L1–3) showed that each aecium consisted of 8.3 cups (SD = 4.8) per mm^2^ of fungal area (*n* = 129). Red line, aecium border; white dots, individual cluster cups; scale bar: 1 mm. **e** Evaluation of scanning electron micrographs illustrated that each cluster cup contained 265 aeciospores (red dots) per layer (*n* = 47), **f** with an average width per layer of 13.9 µm (SD = 2.7; *n* = 52) and **g** average aecial cup length of 374.9 µm (SD = 145.9; *n* = 51). Scale bars: 100, 100, and 500 µm respectively. **h** Scanning electron micrographs of *Pg* aeciospores illustrating their two forms, polyhedral and near spherical. Scale bars: 5 µm. **i** Aeciospores immersed in water were significantly enlarged when compared to those immersed in glycerol. *Pg* aeciospores in glycerol had an average diameter of 13.6 µm (SD = 0.6) and area of 142.3 µm^2^ (SD = 11.1), whereas aeciospores immersed in water had an average diameter of 18.6 µm (SD = 2.0) and area of 262.4 µm^2^ (SD = 59.4). (**p < 0.01; *t*-test). *n* = 60; Scale bars: 20 µm. **j** Transmission electron micrographs show the space between aeciospores was an average of 2.0 µm (SD = 1.2; *n* = 138). White arrowheads, interspore gaps; Scale bar: 20 µm. For all box plots, bars represent median values, boxes signify the upper (Q3) and lower (Q1) quartiles, whiskers are located at 1.5 the inter-quartile range.

We analyzed 165 *Pg* aecia from the abaxial side of *B. vulgaris* leaves using electron microscopy. Each individual aecial cup contained characteristic tightly packed polyhedral chains of aeciospores coated by verrucose and enveloped in a smooth-surfaced peridium that ruptured prior to aeciospore release (Fig. 1a^20^). Aeciospores on the leaf surface were frequently found in rows (Fig. 1b), likely adhered by verrucae from neighboring spores and displaying verrucae plugs that are typical of *Pg* aeciospores (Fig. 1c). Further analysis of 129 aecia from three independent sites showed that each aecium consisted of an average of 40 individual cups (SD = 48) and that each mm^2^ of the fungal area contained an average of 8.3 cups (SD = 4.8) (Fig. 1d; Supplementary Fig. S1a). To determine the total number of aeciospores per cup, we evaluated individual aeciospore diameter in the surface layer of cups prior to ejection (13.9 ± 2.7 µm) and the protrusion of cups from the leaf surface (95.1–680.9 µm). We then estimated the number of spores per layer (265, SD = 57.7) and number of layers per cup (Fig. 1e–g). Cups varied greatly in length, depending on developmental stage and year: 417.9 µm (SD = 133.1) in 2018 and 235.1 µm (SD = 87.4) in 2019 (Supplementary Fig. S1b). Using the average cup length for both years (374.9 µm), we calculated that on average each aecial cup could contain 7111 aeciospores, ranging from 1805 aeciospores for the shortest cup (95.1 µm) and 12,916 aeciospores for the longest cup (680.9 µm). These estimates align with a previous report of 11,000 aeciospores counted in a single *Pg* aecial cup^21^.

Aeciospores were present in two forms: polyhedral prior to release and frequently near-spherical following release on the leaf surface (Fig. 1h). It has been reported that high humidity is required to induce the rounding-off of tightly packed angular aeciospores that supports their ejection^18^. To explore the influence of free water on the rounding-off process, we immersed polyhedral aeciospores in water or glycerol and measured their diameter and area. Aeciospores immersed in water showed a significant increase in diameter (5.0 µm) and area (120.0 µm^2^) (e.g., Supplementary Movie S1) when compared to those immersed in glycerol (*t*-test; *p* = 2.74 × 10^−17^ and *p* = 8.89 × 10^−15^ respectively; Fig. 1i). This increase supports the notion that the availability of free water may be required for absorption as a pre-requisite for spore release, which is consistent with observations of high aeciospore release rates during periods of dew and rainfall^18^.

### Aecia hydration creates a fluid lubrication film that facilitates aeciospore expulsion

**Fig. 2:**
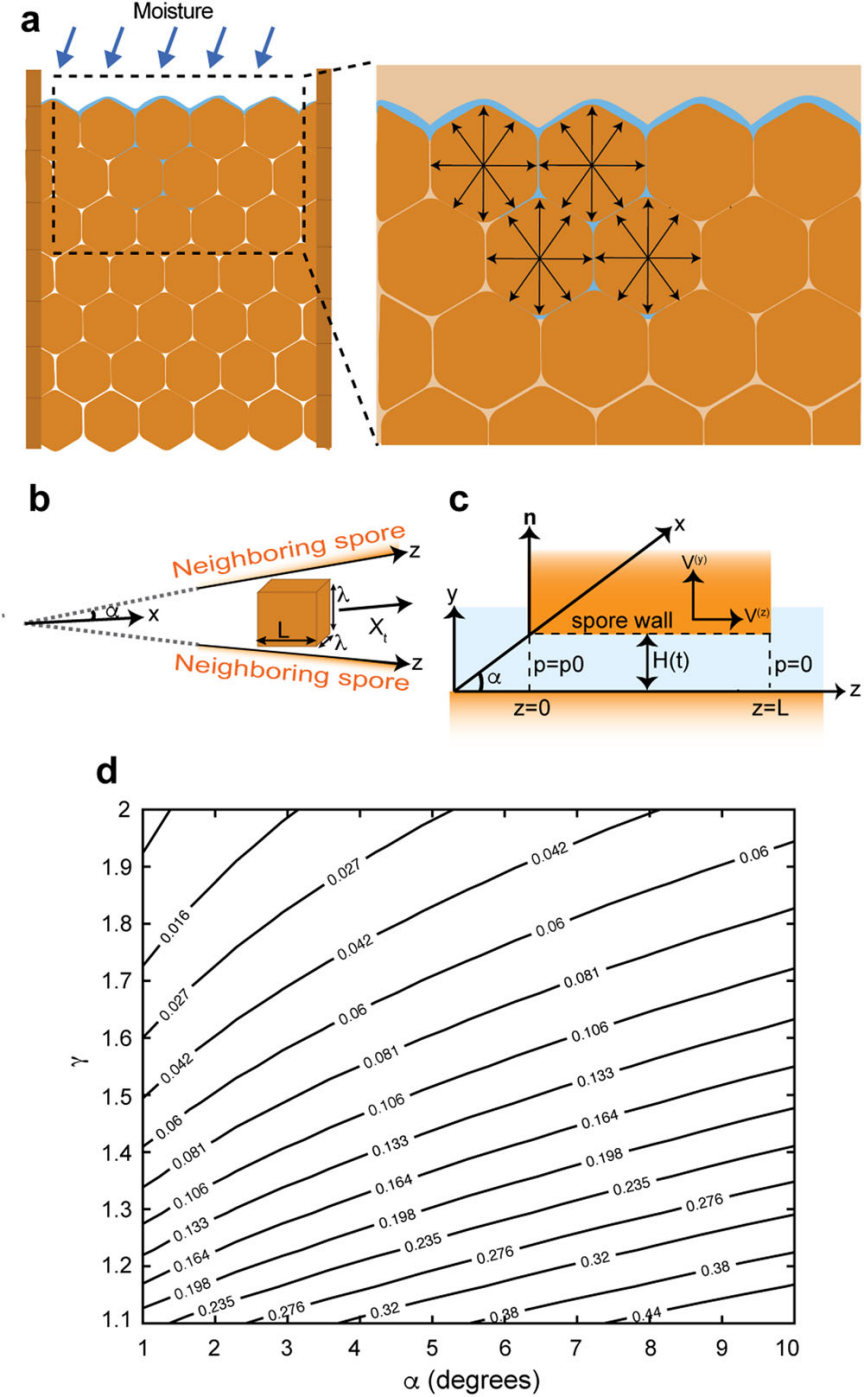
*Pg* aeciospore release speed can be accurately predicted using lubrication theory. **a** Following moisture ingress and hydration of *Pg* aeciospores, the interspore gaps become perfused with water, coating aeciospores with a thin lubrication film. The increase in aeciospore volume through changes in turgidity increases the pressure on neighboring aeciospores, leading to violent ejection. **b** As the mature *Pg* aecium peridium ruptures at the apex it creates an angle of inclination ($\alpha$) that propells aeciospores forward in the direction of the axis of symmetry, i.e., the positive *x* direction. Aeciospores were modeled as cuboids, with sides of length $L\times \lambda \times \lambda$. **c** The edge of the *Pg* aeciospore of length *L* is located at $y=H\left(t\right)$, where $H\left(t\right)$ is the time-dependent gap. The force arising from the film pressure is given by ${{{{{\boldsymbol{P}}}}}}=P{{{{{\boldsymbol{n}}}}}}$, where ${{{{{\boldsymbol{n}}}}}}$ is the unit vector that is normal to the spore face and pointing away from the liquid film and ${p}_{0}$ reflects the heightened pressure acting at the base of each chain of aeciospores as a result of swelling within the aecial cup. The velocity components are given by ${V}^{\left(y\right)}={{{{{\mathrm{d}}}}}H}/{{{{{\mathrm{d}}}}}t}$ and ${V}^{\left(z\right)}=\left({{{{{\mathrm{d}}}}}X}/{{{{{\mathrm{d}}}}}t}\right){{{{{\rm{cos }}}}}}\,{{\alpha }}$. **d** Contour plots illustrate that predicted ejection speeds ranged from 0.02 to 0.5 m s^−1^ for a single aeciospore. Ejection speed was determined by varying the ejection angle $\alpha$ based on approximate values obtained through image analysis (1–11°, i.e., 0.02–0.2 radians).

Aeciospores are tightly packed within aecial cups (Fig. 1j) and expand in volume during hydration, which has been suggested to facilitate aeciospore release by increasing pressure^22^. We reasoned that interspore gaps would become perfused with water following hydration, coating aeciospores with a thin lubrication film. The water lubrication film would reduce the dry state friction in a similar manner to a slider bearing, where the lubrication film acts to reduce contact friction (Fig. 2a). This would ultimately substantially lower the shear force between neighboring aeciospores that opposes translation and mediate the considerable force that is exerted on neighboring aeciospores as they expand. The expansion in aeciospore volume when exposed to water would also reduce interspore gaps, exacerbating the lubrication force. To test whether a model based on these assumptions could successfully describe aeciospore liberation, we developed a model that included a thin lubrication film between aeciospores that would act to distribute pressure as aeciospores expand.

As aecia mature, the peridium ruptures at the apex, creating an incline that generates a component of force on aeciospores that propels them forward in the direction of the axis of symmetry, i.e., the positive *x* direction (Fig. 2b). As the interspore gap is small, we modeled this process using a modified form of the Reynolds lubrication equation (e.g.^23^):

1 $$\frac{1}{12{{\mu }}}\frac{\partial }{\partial z}\left({h}^{3}\frac{\partial p}{\partial z}\right)={V}^{\left(y\right)}-\frac{1}{2}{V}^{\left(z\right)}\frac{\partial h}{\partial z}$$

where $p\left(z,t\right)$ is the pressure in the liquid film, $\mu$ is the dynamic viscosity of water (8.9 × 10^−4^ Pa s), *V*^(*y*)^ and *V*^(*z*)^ are the velocity components of the spore in the *y* and *z* directions respectively (Fig. 2c). The polyhedral aeciospores were approximated as cuboids with the edge of the aeciospore of length *L* located at $y=H(t)$, where $H(t)$ is the time-dependent gap (Fig. 2c). The velocity components are given by ${V}^{(y)}={{{{{\mathrm{d}}}}}}H/{{{{{\mathrm{d}}}}}}t$ and ${V}^{(z)}=({{{{{\mathrm{d}}}}}}X/{{{{{\mathrm{d}}}}}}t){{{{{\mathrm{cos}}}}}}$, where $x=X(t)$ denotes the location of the center of mass of the aeciospore, and the angle $\alpha$ is indicated in Fig. 2b, c and is assumed to be constant. Inserting these into Eq. (1), we have:

2 $$\frac{1}{12{{\mu }}}\frac{\partial }{\partial z}\left({h}^{3}\frac{\partial p}{\partial z}\right)=\frac{{{d}H}}{{{d}t}}-\frac{1}{2}\frac{{{d}X}}{{{d}t}}{{{{{\rm{cos }}}}}}{{\alpha }}\; {{{{{\rm{tan }}}}}}\beta$$

This equation is to be solved for $p$ assuming that ${p=p}_{0}$ at $z=0$ and $p=0$ at $z=L$. Here ${p}_{0}$ reflects the heightened pressure acting at the base of each chain of aeciospores as a result of swelling within the aecial cup. The total hydrodynamic force acting on the face of the aeciospore at $y=H\left(t\right)\,$comprises that due to the fluid pressure and the viscous drag associated with the motion in the film. The force arising from the film pressure is given by ${{{{{\boldsymbol{P}}}}}}=P{{{{{\boldsymbol{n}}}}}}$, where ${{{{{\boldsymbol{n}}}}}}$ is the unit vector that is normal to the spore face and pointing away from the liquid film (Fig. 2c), and

3 $$P={{\lambda }}\int_{0}^{L}p{dz}={{\mu }}{{\lambda }}\frac{{L}^{3}}{{H}^{3}}\left(-\frac{{{d}H}}{{{d}t}}\right).$$

Where $\lambda$ is the length of the aeciospore in the transverse direction. The viscous drag force on the aeciospore face is given by ${{{{{\boldsymbol{D}}}}}}{{{{{\boldsymbol{=}}}}}}-D{{{{{{\boldsymbol{e}}}}}}}_{z}$, where ${{{{{{\boldsymbol{e}}}}}}}_{z}$ is the unit vector pointing in the positive ${{{{{\rm{z}}}}}}$ direction, and

4 $$D={{\mu }}{{\lambda }}\int_{0}^{L}{\frac{\partial u}{\partial y}}_{y\,=\,H},$$

where the velocity in the film is

5 $$u=\frac{1}{2{{\mu }}}y\left(y-H\right)\frac{\partial p}{\partial z}+\frac{y}{H}\frac{{{d}X}}{{{d}t}}{{{{{\rm{cos }}}}}}{{\alpha }}.$$

Accounting for each of the four faces that oppose aeciospores in adjacent chains and including the pressure force and viscous drag, the total hydrodynamic force acting on the aeciospore in the *x* direction, is:

6 $${{{\lambda }}}^{2}{p}_{0}+4F$$

where ${{{\lambda }}}^{2}{p}_{0}$ arises due to the pressure ${p}_{0}$ acting on the bottom face of the aeciospore, and

7 $${{{{{\rm{F}}}}}}\equiv \left({{{{{\boldsymbol{P}}}}}}+{{{{{\boldsymbol{D}}}}}}\right)\cdot {{{{{{\boldsymbol{e}}}}}}}_{x}=P{{{{{\rm{sin }}}}}}{{\alpha }}-D{{{{{\rm{cos }}}}}}\propto ,$$

where ${{{{{{\boldsymbol{e}}}}}}}_{x}$ is the unit vector in the $x$ direction. Further details regarding the pressure force calculations are provided in Supplementary Note **1**. This analysis illustrates that the presence of a thin lubrication film between aeciospores could mediate the substantial force required to facilitate liberation.

### Aeciospore release speed can be predicted using lubrication theory

To further determine if aeciospore liberation can be accurately modeled using lubrication theory, we generated theoretical spore release speeds using this approach. We accounted for the force acting on all four faces that oppose neighboring aeciospores by applying Newton’s second law of motion and obtained the equation of motion for the aeciospore center of mass

8 $$\frac{{{d}}^{2}X}{{d}{t}^{2}}+A\frac{{{d}X}}{{{d}t}}+B=0,$$

where:

$$A=\frac{4{{\mu }}{{\lambda }}}{m}\frac{L}{H}{{{{{{\rm{cos }}}}}}}^{2}{{\alpha }},$$

9 $$B=-\frac{4{{\upmu }}{{\lambda }}}{m}\frac{{L}^{3}}{{H}^{3}}\left(-\frac{{{d}H}}{{{d}t}}\right){{{{{\rm{sin }}}}}}{{\alpha }}-\frac{2{{\lambda }}}{m}\left(L{{{{{\rm{sin }}}}}}{{\alpha }}+2{{\lambda }}+H{{{{{\rm{cos }}}}}}{{\alpha }}\right){p}_{0}$$

and $m$ is the mass of the aeciospore. We hypothesize that as aeciospores expand, the interspore gap closes following a power law so that

10 $$H\left(\tau \right)={H}_{0}{\left(1+\kappa \tau \right)}^{-1/\left(\gamma -1\right)}$$

for suitable chosen constants ${{{{{\rm{\kappa }}}}}}$ and ${{{{{\rm{\gamma }}}}}}$, where ${H}_{0}$ is the size of the initial gap determined by TEM image analysis (1.96 × 10^−6^ m; Fig. 1j; Supplementary Fig. S2). The key parameters in the model are the power law exponent ${{{{{\rm{\gamma }}}}}}$, and the angle of inclination $\alpha$ (Fig. 2b, c), for which a range of $\alpha$ values were approximated based on observations from image analysis (1–11°, i.e., 0.0–20.2 radians). Using this model, the observed values for $\alpha$ and a range of values for the power law exponent ${{{{{\rm{\gamma }}}}}}$, single aeciospores were predicted to be ejected at speeds ranging from 0.02 to 0.5 m s^−1^ (Fig. 2d). To demonstrate the robustness of these predictions with respect to changes in the parameters, we assessed the ejection speed whilst varying the initial gap ${H}_{0}$ for the sample power law exponent $\gamma =1.25$ for three different ejection angles ${{{{{\rm{\alpha }}}}}}$ (2°, 5° and 8°). In all three cases, predicted ejection speeds remained close to those initially projected, e.g., within the range of 0.21–0.4 m s^−1^ for the case of a 5° ejection angle when ${H}_{0}$ varies over the range of 0.5–3 $\upmu$m (Supplementary Fig. S3). Furthermore, as pressure builds the interspore gap may close and the lubrication pressure could become large enough to drive fluid in the negative *z* direction towards the base of the aecial cup as aeciospores are ejected. To account for this, we also adjusted the base pressure ${p}_{0}$ dynamically to prevent backflow. Ejection velocities determined following this supposition, were broadly in line with the above predictions (Supplementary Fig. S4). Further details regarding the theoretical model of aeciospore ejection are provided in Supplementary Note 1.

### Aeciospores are mechanically released to escape the leaf boundary layer

**Fig. 3:**
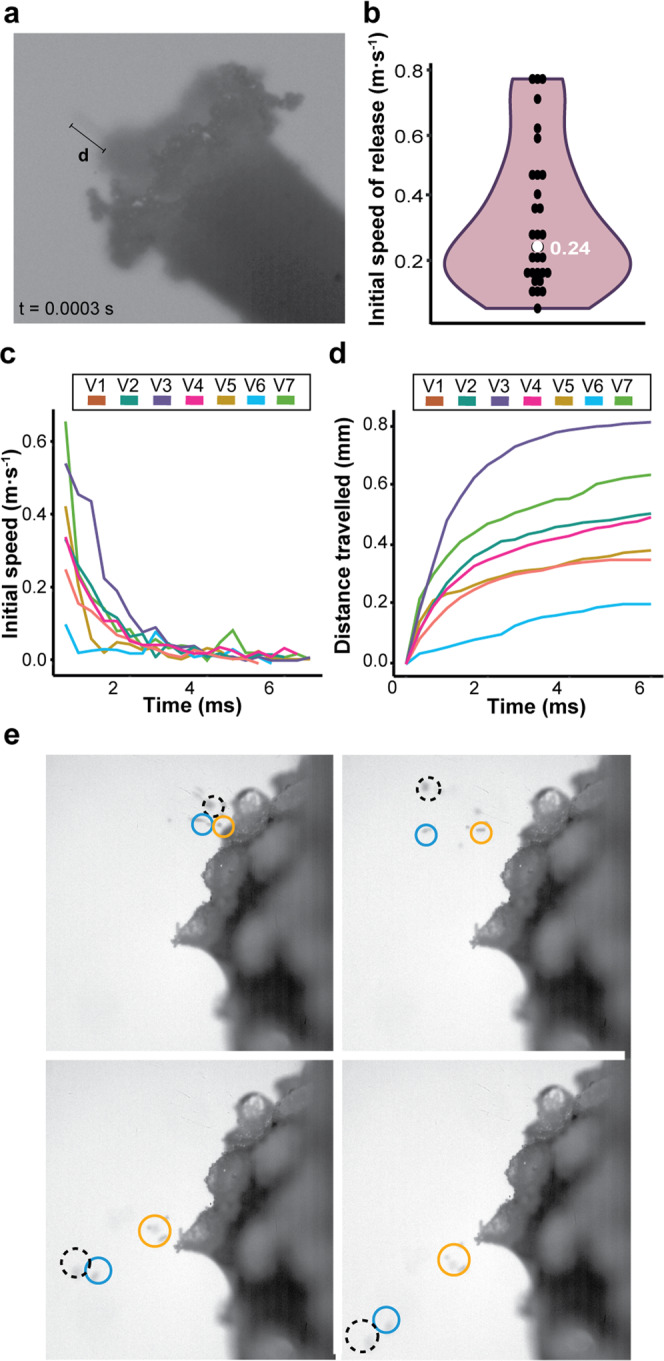
Pg aeciospores released in clusters achieve greater distances. **a** *Pg* aeciospore ejection was recorded using high-speed videography at 3000 frames per second (fps). d, distance; t, time; s, seconds. **b** The initial ejection speed of *Pg* aeciospores ranged from 0.053 to 0.754 m s^−1^ (median 0.24 m s^−1^; *n* = 31). **c**, **d** After ejection, aeciospore speed rapidly decreased over time. Aeciospore speed of discharge was measured frame by frame until aeciospores began to decelerate as drag force was exerted. V1–7 represent seven independent aeciospore release events. **e** Aeciospores released in groups achieved greater distances. Single frames from high-speed videography of *Pg* aeciospore discharge. Black broken circle highlights a cluster of aeciospores being released, blue and orange circles highlight spores released in chains.

To establish the true ejection speed and trajectory of aeciospore release, we assessed spore release from 31 aecia with high-speed videography at 3000 frames per second (fps), which was the maximum fps that could be achieved whilst still maintaining illumination across the aecial cup. (Supplementary Fig. S5; Supplementary Movies S2–3). Initial ejection speed was determined from the first frame following release (0.0003 s; Fig. 3a, b) and observed from 0.053 to 0.754 m s^−1^, with a median of 0.24 m s^−1^. To assess initial aeciospore trajectory, speed over time was measured frame by frame until aeciospores began to decelerate as drag force was exerted^23^ and the distance traveled reached a sharp plateau, where speed approached 0 m s^−1^ (Fig. 3c–e and Supplementary Fig. S6). These initial ejection speeds are consistent with those predicted in the theoretical model, providing further support for our hypothesized moisture-induced mechanism of aeciospore release.

### The release of aeciospores in clusters may enhance long-distance dispersal

**Fig. 4:**
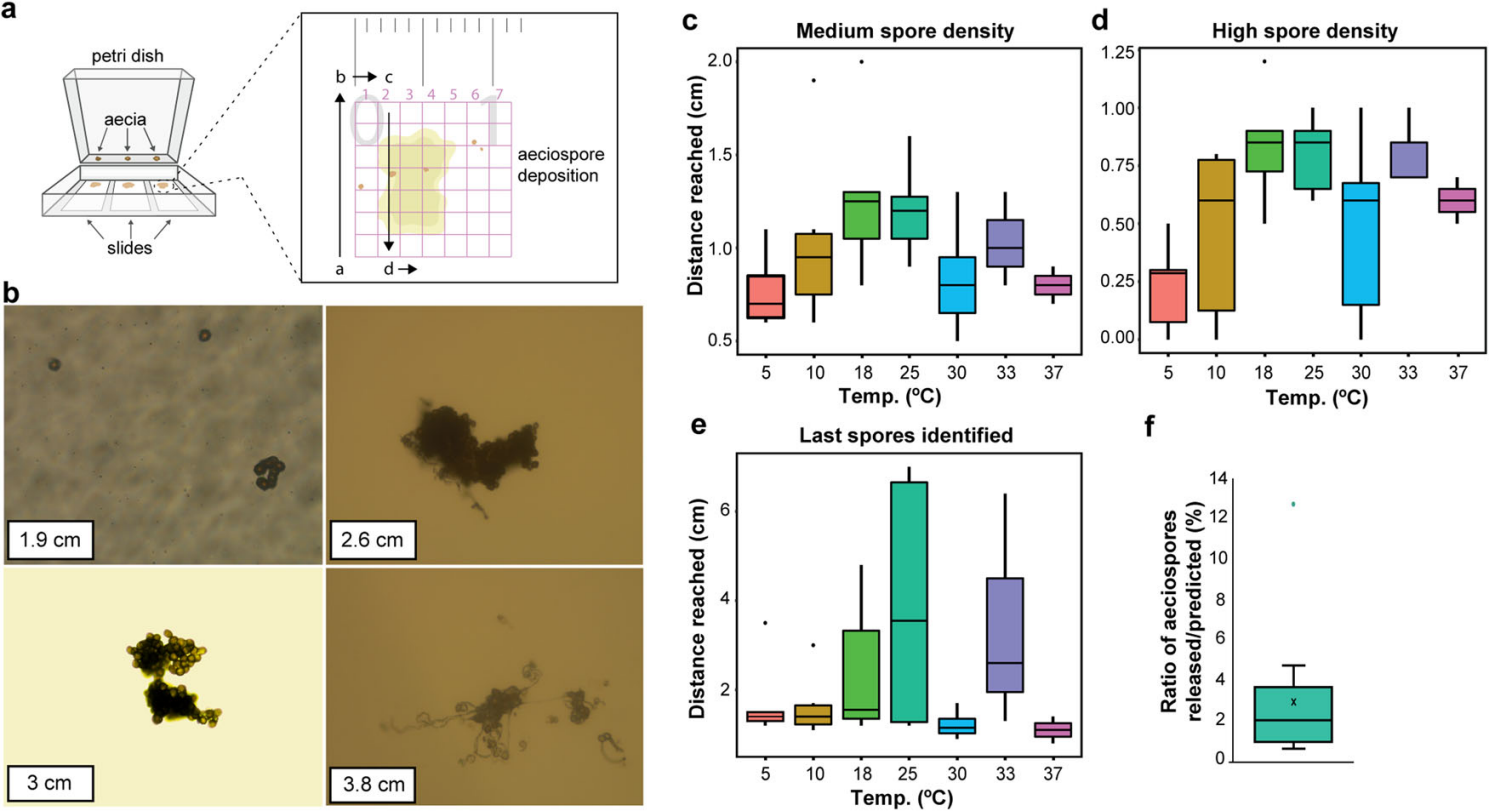
Changes in temperature did not alter the rate of aeciospore release. **a** The distance traveled by aeciospores following ejection was determined. *B. vulgaris* leaf fragments containing a single *Pg* aecium were attached to the wall of a 100 mm square petri dish and aeciospore discharge distances measured by analysis of spore quantity across a series of adjacent microscope slides with a thin (2%) water agar layer. Aecial cups were incubated at 18 °C and assessments made after 18 h. **b** Representative micrographs of aeciospores identified at different distances from the aecium (1.9, 2.6, 3, and 3.8 cm) illustrate that aeciospores found more than 2 cm from the aecium were commonly found in clusters. **c**–**e** Alterations in temperature did not influence the distance aeciospores traveled. Aeciospore release was evaluated under 5, 10, 18, 25, 30, 33, and 37 °C, with no significant difference found (*n* = 6, Tukey’s HSD; *p* < 0.01). **f** The number of aeciospores released was much lower than the predicted full potential. Aeciospore content (“predicted”) per aecium were estimated by measuring the fungal mass and compared to those recorded as released (“released”). *n*  = 3; For all box plots, bars represent median values, boxes signify the upper (Q3) and lower (Q1) quartiles, whiskers are located at 1.5 the inter-quartile range.

To examine the distance aeciospores could travel in a closed environment, aecial cups were incubated for 18 hours in darkness at 18 °C and slides positioned adjacent to capture released aeciospores (Fig. 4a). In all experiments, high spore density (>100 spores/0.2  mm^2^) was observed close to the source, reaching a maximum distance of 0.50–1.20 cm (median 0.85 cm), progressively becoming more dispersed. The maximum distance aeciospores reached varied, from 1.20 to 4.80 cm (median 1.55 cm). However, aeciospores at greater distances (more than 2 cm), were consistently found in clusters (Fig. 4b). The maximum distance aeciospores traveled was used to estimate the initial speed of release^24^. Aeciospore release speed was determined using maximum distances from high spore density (>100 spores/0.2 mm^2^), medium spore density (10–50 spores/0.2 mm^2^), and final maximum distance (last spore found) (Supplementary Table S1, Fig. S7). When using the maximum distance, release speeds reached tens of meters per second, which was 100 times greater than that observed using high-speed videography. However, as aeciospores identified at greater distances were consistently found in clusters, this would increase the mass-air drag (m/ζ ratio), thereby facilitating greater distances^25^. Using the lubrication model, we varied the size of aeciospore diameter to simulate the release of a cluster or chain of aeciospores. When the diameter was increased, the predicted speeds also increased (Supplementary Fig. S3). Thus, our theoretical model predicts that the release of aeciospores in clusters or chains could enhance ejection speeds, which may act to amplify the initial distance traveled, ensuring they exit the leaf boundary layer and enter air currents.

### Alterations in temperature do not influence the rate of aeciospore release

To investigate the effects of temperature on the rate of aeciospore release, we monitored release at 5 °C, 10 °C, 18 °C, 25 °C, 30 °C, 33 °C, and 37 °C. The maximum distance aeciospores reached was highly variable (1.22–7.12 cm), but no statistically significant differences (*p* < 0.01) between temperatures were observed (Fig. 4c–e). We also quantified the number of aeciospores released under each temperature; the cumulative number of aeciospores released showed a normal distribution with most aeciospores falling adjacent to the aecium (Supplementary Fig. S8). The area of fungal mass per aecium was measured in each case to determine the expected number of aeciospores that could be released (Supplementary Table S2). The ratio between the expected and observed number of aeciospores varied between and within temperatures, with an average of only 2.83 % of expected aeciospores released across temperatures (Fig. 4f). We conclude that this range of temperatures had minimal impact on the rate of aeciospore expulsion, although the results highlight that the number of aeciospores released was much lower than the theoretical potential. This difference may reflect a need for successive release events to expel the full complement of aeciospores within a single aecium.

### Estimating long-distance aeciospore dispersal

**Fig. 5:**
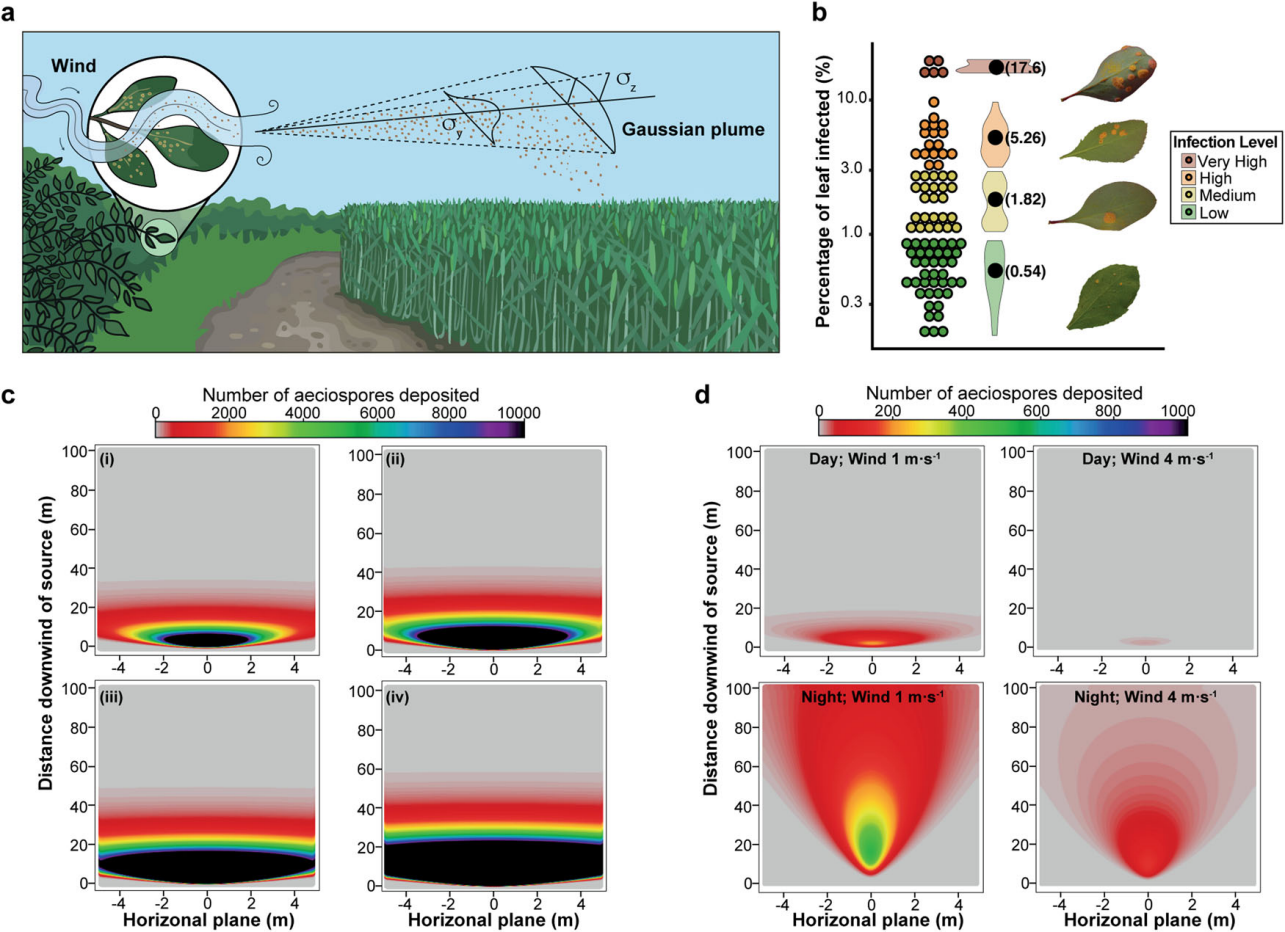
Aeciospore dispersal gradient can be simulated using a Gaussian Plume (GP) model. **a** A GP model was used to simulate how far *Pg* aeciospores may travel following release. **b** *B. vulgaris* leaves were divided into four categories reflecting their predicted level of *Pg* infection. The percentage of fungal area was estimated for each group resulting in: 0.54% (low), 1.82% (medium), 5.26% (high) and 17.6% (very high). *n* = 85. **c** Density plots illustrate that the distance aeciospores traveled varied depending on source strength. Four source strength values were used to reflect the four levels of *Pg* infection: 316,121 (i), 1,065,445 (ii), 3,079,254 (iii) and 10,303,206.9 (iv) aeciospores. **d** Density plots show how environmental conditions considerably influence aeciospore dispersal. Upper graphs represent day conditions (620 W/m^2^ solar radiation and 10% cloud cover) lower graphs represent night conditions (no solar radiation and 50% cloud cover). Wind speed was set at 1 m s^−1^ (left graphs) and 4 m s^−1^ (right graphs).

To estimate the concentration of aeciospores at each point downwind from the source (i.e., a *B. vulgaris* bush) we used a Gaussian Plume (GP) model and determined the potential source strength using predictions of aecia spore content (Fig. 5a). To estimate the concentration of aeciospores ($C$) that would be deposited over adjacent gramineous host plants and thus could potentially start an infection, the following equation from^26^ was used:

11 $$C=\frac{{Q}_{{{{{{\rm{eff}}}}}}}}{2\pi u{{\sigma }_{y}\sigma }_{z}}\cdot {e}^{\left(-\frac{{y}^{2}}{2{{\sigma }_{y}}^{2}}\right)}\cdot {e}^{\left(-\frac{{H}^{2}}{2{{\sigma }_{z}}^{2}}\right)}{e}^{\left(-\frac{{\left(H-2d\right)}^{2}}{2{{\sigma }_{z}}^{2}}\right)}$$

The formula gives the concentration *C* at a point at ground level (*x,y*) due to a source of strength ${Q}_{{{{{{\rm{eff}}}}}}}$ located at a height H above the ground in a wind speed *u* (m/s). Here *x* and *y* measure distance in the mean wind direction and in the transverse crosswind direction respectively. Here$\,{Q}_{{{{{{\rm{eff}}}}}}}={Q}_{0}\times {F}_{d}\times {F}_{s},$ where F_d_ and F_s_ are the fractions of initial source strength that will remain viable after deposition and solar radiation respectively, both factors affected by *x* and previously defined at^27^. The formula accounts for the presence of the ground by incorporating an effective image source located beneath ground level; the parameter *d* accounts for a vertical shift in the wind profile^27^. The standard deviations ${\sigma }_{y}$ and ${\sigma }_{z}$ measure the spread of plume in the crosswind (*y*) and vertical (*z*) directions respectively and are also affected by *x*.

We measured aecia coverage across 85 *B. vulgaris* leaves infected with *Pg* and divided infection levels into four intensity categories: low (<1% of leaf covered by aecia), medium (1–3%), high (3–10%) or very high (>10%). The percentage of fungal area per leaf was estimated for each group resulting in 0.54% (low), 1.82% (medium), 5.26% (high) and 17.6% (very high) (Fig. 5b). These values were then used to estimate the source strength at each level of intensity, i.e., the total number of aeciospores with the potential for release per leaf (Fig. 5c). This included an approximation of the number of *B. vulgaris* leaves infected within a single bush (maximum 900 leaves), estimated from evaluation of available literature^28,29^.

**Fig. 6:**
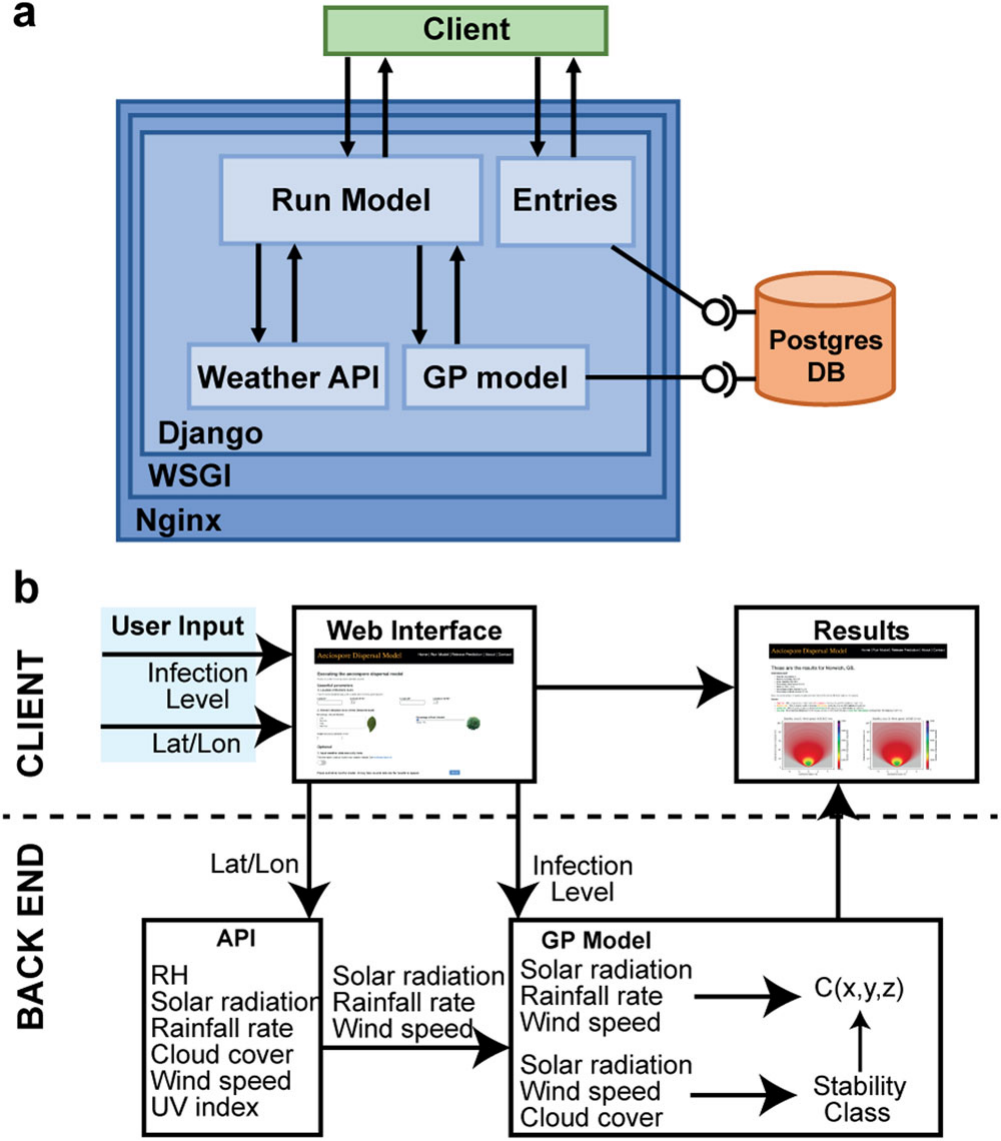
The Gaussian Plume (GP) aeciospore dispersal model was integrated into a webapp interface. **a** Diagram illustrating the core components of the webapp developed using Django, with a postgres database and nginx web server. WSGI web server gateway interface, API application programming interface, DB database. **b** Illustration of the utility of the aeciospore dispersal model webapp. When a request is submitted, the location (latitude/longitude) is input into an API that gathers weather data and inputs this into the GP model with the infection level provided by the user. The results are returned illustrating the density of aeciospores at various distances from a *Pg*-infected *B. vulgaris* bush for the current environmental conditions in the given location. RH relative humidity.

Due to variability in source strength and its profound impact on aeciospore dispersal^30^, alongside environmental factors, assessing the risk posed by specific barberry bushes varies depending on conditions and location (Fig. 5d). Therefore, we incorporated our model into a web interface where the location of a *B. vulgaris* bush and its infection level can be entered, and an application programming interface (API) collects current weather data and calculates the concentration of aeciospores that would be found at varying distances from the source (Fig. 6a, b;^31^). This tool can now be used to aid in risk assessments of *Pg*-infected *B. vulgaris* bushes that are in close proximity to potential *Pg*-host crop plants.

## Discussion

Fungi exhibit a range of spore discharge processes. Here, we developed a theoretical model of the ejection mechanics of aeciospore release for *Pg* using lubrication theory. During periods of dew or rainfall, the inner thin wall of peridial cells in *Pg* aecidioid aecia expands to a greater degree than the thick outer wall, causing the peridium to rupture^32^. This rupture is followed by moisture ingress, hydrating and rounding-off the constrained angular aeciospores^18,19^. We reasoned that adhesive forces of the verrucae that adhere neighboring aeciospores in the aecium would be suddenly overcome as they expand, while moisture ingress coats rounded aeciospores in a thin lubrication film that would facilitate violent active expulsion from ruptured aecial cups, similar to a squeeze catapult^2^. Using our theoretical model, we predicted ejection speeds of single aeciospores between 0.02 and 0.5 m s^−1^, consistent with observations using high-speed videography where aeciospores reached speeds averaging 0.24 m s^−1^. This speed is lower than that reported for other discharge processes such as the surface-tension energized catapult mechanism for Basidiomycetes (launching spores at speeds from 0.53 to 1.1 m s^−1^) and the squirt guns pressurized by osmosis that are common in the Ascomycetes (spore speeds of 9–21 m s^−1^)^4,33^. Furthermore, we observed that aeciospores were frequently released in clusters, a process previously termed ‘*Pg* cluster-bombs’^21^. Here we found that aeciospore cluster release enhanced initial ejection speeds and ejection distances with ‘*Pg* cluster-bombs’ reaching a maximum of 7.12 cm. Following discharge and exit of the leaf boundary layer, groups of aeciospores would likely disassociate to enhance subsequent wind transmission.

Aecia development is tightly governed by temperature; aecial cups appear much later and are more elongated when *B. vulgaris* leaves displaying *Pg* pycnia are incubated at temperatures below 9 °C compared to temperatures over 24 °C^34^. This is consistent with the slightly elongated aecia we observed in 2018 compared to 2019, coinciding with a cooler early spring in the UK in 2018^35^. Although we determined here that the influence of temperature on aeciospore ejection was negligible, variability in aecial cup extension would have a significant effect on subsequent source strength. Such biological variability is notoriously difficult to accurately factor in dispersal modeling^30^. Accordingly, previous attempts have generated wildly different estimates for *Pg* aeciospore source strength that equate to either 1 or 64 billion aeciospores per *B. vulgaris* bush^15,22^.

Here, we estimated average source strength per aecium through detailed microscopic analysis and discharge measurements. We assessed aecial coverage across *B. vulgaris* leaves to define four broad intensity levels, approximated the number of infected leaves in a single *B. vulgaris* bush and employed a GP model to simulate aeciospore dispersal gradients. GP models do have inherent limitations due to their underlying assumptions, particularly that the shape of the plume is everywhere assumed to conform to a Gaussian distribution and that it assumes a unique source point^36^. However, for incorporation into a web interface the simplicity and thereby low computational power of the GP model made it the most suitable^37^. This approach provides the first model of aeciospore dispersal gradients that also considers the variability in infection levels and provides a range of source strength approximations within an easily executable web interface. Our interface can be employed by policy makers, conservationists, and farmers to guide local decisions regarding planting of *Pg*-susceptible *Berberis* species and disease management.

Here, we have started to unravel the long-standing mysteries surrounding the aeciospore dispersal mechanism. Attempts to rationalize the process of discharge in the 1920s suggested the verrucae plugs that adorn the surface of aeciospores from species such as *Pg* may play a role in the liberation mechanism^19,38^. Accordingly, it was suggested that aeciospores tightly packed in the aecium are indented by verrucae plugs and during the rounding-off process these plugs may act to enhance ejection speeds, similar to compression of a tennis ball over a marble^38^. However, this theory does not account for species where aeciospores lack verrucae plugs. In our model, although these adornments could act to enhance the angularities of densely packed aeciospores, moisture ingress and subsequent changes in turbidity alongside the presence of a lubrication film are the most critical components. In the future, assessing the speed of aeciospore discharge in species that lack verrucae plugs such as *P. coronata* var. *avenae*^32^ could help clarify their contribution to the process. Our model and new knowledge regarding *Pg* aeciospore ejection mechanics and dispersal now provide the means to guide general legislation regarding *B. vulgaris* planting so that this species can be maintained as an important element of biodiversity without threatening food supply. This will be of particular value in western Europe, where legislation governing *Berberis* planting has long-since lapsed and *B. vulgaris* is increasing in prevalence, concomitant with an increase in wheat stem rust outbreaks after many decades of absence^11^. Our results will thus inform detailed epidemiological risk models to protect small-grain crops from early-season inoculum and help reduce the future severity of *Pg* epidemics originating on *Berberis*.

## Materials and methods

### Sample collection, aeciospore expansion analysis, and high-speed videography of aeciospore release

*B. vulgaris* leaves that displayed yellow, tube-like aecial structures on their abaxial side, typical of cluster cup rust caused by *Pg* were collected in four locations (Brandon, Babraham, Mansfield and around Kelso) in the UK throughout the spring and summer in 2018–2020. *B. vulgaris* leaves were initially incubated at 4 °C prior to use. Aecia were induced to release aeciospores by suspending the leaf fragment over water and spraying with additional water mist directly onto the cluster cups. To quantify the increase in aeciospore size following water intake, released aeciospores were air dried in silica gel overnight and mounted in water or 100% glycerol on microscope slides. Specimens were examined under a Zeiss LSM780 confocal microscope (Zeiss, Germany) using a Plan NeoFluar 25x/NA 0.8 immersion objective with an excitation wavelength of 405 nm (30 mW diode laser) and emission bandwidth of 410–468 nm. Image stacks were collected with a GaAsP detector array and a pinhole of 1.1 AU. Voxel size was 71 nm in x/y and 510 nm in z. Images were analyzed using ImageJ, with three measurements taken and averaged to determine aeciospore diameter: diagonal, vertical, and horizontal. High-speed videography recordings of aeciospore expulsion were performed using a Photron Fastcam SA-X2 mono high-speed camera (Photron, USA) with a Navitar 12× zoom lens at maximum magnification (3000 fps at 1025 × 1024 pixels resolution). Extra illumination was provided by two LED beam lights and images captured using the Photron Fastcam Viewer software. Discharge speed was determined by analysis of the first frame of the recording using ImageJ.

### Establishing the distance reached by aeciospores following discharge

*B. vulgaris* leaf fragments containing a single *Pg* aecium were briefly suspended over water and then attached to the wall of a 100 mm square petri dish with Vaseline® (Unilever UK Limited, UK). Aeciospore discharge distances were measured by analysis of spore quantity across a series of numbered microscope slides with a thin (2%) water agar layer, placed in a straight path from the aecium. Aecial cups were sprayed with a fine water mist to increase humidity and plates incubated at 5 °C, 10 °C, 18 °C, 25 °C, 30 °C, 33 °C or 37 °C for 18 h. Six biological replicates were performed for distance measurements and three for quantifying aeciospore release for each temperature. Deposited aeciospores were observed under an inverted Leica DMi1 microscope (Leica, UK) at ×10 or ×20 magnification, representing an area of 1 × 1.5 mm^2^ or 0.5 × 0.75 mm^2^ respectively. To estimate the total number of aeciospores released per aecium, images were analyzed using a python script and then manually checked for confirmation. A Tukey’s honestly significant difference (HSD) post hoc test was performed to compare release distances.

### Cryo-scanning electron microscopy of *Pg* aeciospores and fungal structures

Samples of *B. vulgaris* leaf fragments with *Pg* aecia were first mounted on aluminum stubs using Tissue Tek^R^ (BDH Laboratory Supplies, UK). The stubs were then immediately plunged into liquid nitrogen slush at approximately −210 °C to cryopreserve the material. Samples were transferred onto the cryostage of an ALTO 2500 cryotransfer system (Gatan, UK) attached to a Zeiss Supra 55 VP FEG scanning electron microscope (Zeiss SMT, Germany) or the same type of cryo-system on a FEI Nova NanoSEM 450 (FEI, The Netherlands). Sublimation of surface frost was performed at −95 °C for ~3 min before the samples were sputter coated with platinum for 2 min at 10 mA, at colder than − 110 °C. After sputter-coating, the samples were moved onto the cryostage in the main chamber of the microscope, held at −125 °C. The samples were imaged at 3 kV and digital TIFF files stored. Images were analyzed using ImageJ.

### Transmission electron microscopy of *Pg* aecia

*B. vulgaris* leaf fragments with *Pg* aecia were placed in a solution of 2.5% (v/v) glutaraldehyde in 0.05 M sodium cacodylate (pH 7.3), vacuum infiltrated, and incubated at room temperature (RT) overnight, before three successive 10-min washes in 0.05 M sodium cacodylate. Samples were post-fixed in 1% (w/v) OsO_4_ in 0.05 M sodium cacodylate for one hour at RT. The osmium fixation was followed by three, 15-min washes in distilled water before beginning an ethanol dehydration series (30, 50, 70, 95, and 100% ethanol), for approximately one hour in each solution. Once fully dehydrated, samples were gradually infiltrated with LR White resin (London Resin Company, UK) by successive changes of resin-ethanol mixtures at RT, as follows: 1:1 for 1 h, 2:1 for 1 h, 3:1 for 1 h, 100% resin for 1 h then 100% resin for 16 hours. After a fresh resin change for a further 8 h, samples were transferred into gelatin capsules full of fresh LR White and placed at 60 °C for 16 h to polymerize. The material was sectioned with a diamond knife using a Leica UC6 ultramicrotome (Leica, UK). Ultrathin sections of ~90 nm were picked up on formvar and carbon coated 200 mesh copper grids. Sections were stained with 2% (w/v) uranyl acetate for 1 h and 1% (w/v) lead citrate for 1 min, washed in distilled water, and then air dried. Grids were viewed in a FEI Talos F200C transmission electron microscope (FEI UK Ltd, UK) at 200 kV and imaged using a Gatan OneView 4 K × 4 K digital camera (Gatan, UK) to record DM4 files.

### Web interface design and availability

The web interface was developed using python3 and Django (version 3.0.2)^39^. Weather parameters were gathered using the weatherbit API^40^. A Django form was established to obtain the input values for the location (latitude and longitude), percentage of infection, and height of the source. All templates were designed using HTML and CSS (bootstrap-4.0.0) for visual aspects. The interactive options were coded using jQuery (version 1.12.0). The specific packages can be found in the *base.html* template in GitHub. The aeciospore dispersal model web interface is centrally hosted at the John Innes Centre and available at: www.aeciospore-dispersal-model.com.

### Statistics and reproducibility

A two-tailed Student’s *t* test was used to evaluate the significance of differences in (i) diameter and area of aeciospores incubated in water or glycerol (*n* = 60), (ii) cluster cup length between aecia collected in 2018 (*n* = 39) and 2019 (*n* = 12), and (iii) the ratio between expected and observed number of aeciospores released between temperatures (*n* = 3, per temperature). A Tukey’s HSD post hoc test was performed to compare the distance aeciospores reached at different temperatures at different densities (medium and high density and last aeciospore identified; *n* = 6). All statistical tests were conducted in R and Excel, with further details of statistical analyses provided in Supplementary Data 1. All samples were used in the study without exclusion and sample size and repetition in each experiment was largely determined by the availability of material.

### Reporting summary

Further information on research design is available in the Nature Research Reporting Summary linked to this article.

## Supplementary information

Supporting Information

Description of Additional Supplementary Files

Supplementary Movie S1

Supplementary Movie S2

Supplementary Movie S3

Supplementary Data 1

Supplementary Data 2

Reporting Summary

## Data Availability

The data that support the findings of this study are available in the supplementary material of this article (Supplementary Data 2).

## Code availability

Python scripts developed to quantify the number of aeciospores per layer, number of aecial cups per aecium, and number of aeciospores released are available on GitHub^41^ and zenodo^42^. The code for building the front-end of the aeciospore dispersal model web interface and back-end database is also available on GitHub^43^ and zenodo^44^.

## References

[1] Bebber DP, Gurr SJ (2015). Crop-destroying fungal and oomycete pathogens challenge food security. Fungal Genet. Biol..

[2] Sakes A (2016). Shooting mechanisms in nature: a systematic review. PLoS ONE.

[3] Lagomarsino Oneto D, Golan J, Mazzino A, Pringle A, Seminara A (2020). Timing of fungal spore release dictates survival during atmospheric transport. Proc. Natl Acad. Sci. USA.

[4] Fischer MW, Stolze-Rybczynski JL, Cui Y, Money NP (2010). How far and how fast can mushroom spores fly? Physical limits on ballistospore size and discharge distance in the Basidiomycota. Fungal Biol..

[5] Meyer M (2017). Quantifying airborne dispersal routes of pathogens over continents to safeguard global wheat supply. Nat. Plants.

[6] Singh Saharan, G., Verma, P. R., Meena, P. D. & Kumar, A. in *White Rust of Crucifers: Biology, Ecology and Management* (Springer, 2014).

[7] Liu, F. et al. Asymmetric drop coalescence launches fungal ballistospores with directionality. *J R Soc Interface* **14**, 10.1098/rsif.2017.0083 (2017). 10.1098/rsif.2017.0083 PMC5550963 28747394

[8] Perdomo-Sánchez O, Piepenbring M (2008). A new species of *Puccinia* (Pucciniales, Basidiomycota) and new records of rust fungi from Panama. Mycological Prog..

[9] Szabo, L. J., Cuomo, C. A. & Park, R. F. in *Genomics of Plant-Associated Fungi: Monocot Pathogens* (eds Dean, R. A., Lichens-Park, A. & Kole, C.) 177–196 (Springer Berlin Heidelberg, 2014).

[10] Kim S, Park H, Gruszewski HA, Schmale DG, Jung S (2019). Vortex-induced dispersal of a plant pathogen by raindrop impact. Proc. Natl Acad. Sci. USA.

[11] Saunders DGO, Pretorius ZA, Hovmoller MS (2019). Tackling the re-emergence of wheat stem rust in Western Europe. Commun. Biol..

[12] Stakman, E. C. *Barberry Eradication Prevents Black Rust in Western Europe* (United States Department of Agriculture, Washington D.C., 1923).

[13] Peterson, P. D. *Stem Rust of Wheat: From Ancient Enemy to Modern Foe*. (Amer Phytopathological Society Press, 2001).

[14] Roelfs, A. P. in *Diseases, Distribution, Epidemiology, and Control* (eds Roelfs, A .P. & Bushnell, W. R.) 403–434 (Academic Press, 1985).

[15] Stakman, E. C., Melander, L. W. & Fletcher, D. G. Barberry Eradication Pays. in *Minnesota State Department of Agriculture Bulletin 55* (St. Paul, 1927).

[16] Lambert EB (1929). The relation of weather to the development of stem rust in the Mississippi Valley. Phytopathology.

[17] Van Arsdel, E. P., Conklin, D. A., Popp, J. B. & Geils, B. W. in *Proc. First IUFRO Rusts of Forest Trees Working Party Conference* Vol. 712, 275–283 (Finnish Forest Research Institute, Research Papers, Saariselka, Finland, 1998).

[18] Kramer CL, Pady SM, Clary R, Haard R (1968). Diurnal periodicity in aeciospore release of certain rusts. T Brit Mycol. Soc..

[19] Ingold, C. T. *Fungal Spores: Their Liberation and Dispersal*. (Clarendon Press, 1971).

[20] Sato T, Sato S (1985). Morphology of aecia of the rust fungi. T Brit Mycol. Soc..

[21] Coons GH (1910). Researches on fungi. Science.

[22] Buller, A. H. R. in *Researches on Fungi* Vol. V (Hafner Publishing, 1958).

[23] Batchelor, G. K. *An Introduction to Fluid Dynamics*. (Cambridge University Press, 1967).

[24] Pringle, A., Brenner, M. P., Fritz, J., Roper, M. & A., S. in *The Fungal Community: Its Organization and Role in the Ecosystem* (eds Dighton, J. & White, J. F.) 309–320 (CRC, 2017).

[25] Lacey J (1991). Aggregation of spores and its effect on aerodynamic behavior. Grana.

[26] Spijkerboer HP (2002). Ability of the Gaussian plume model to predict and describe spore dispersal over a potato crop. Ecol. Model.

[27] Aylor DE (1999). Biophysical scaling and the passive dispersal of fungus spores: relationship to integrated pest management strategies. Agr. For. Meteorol..

[28] Khan T (2014). Estimation of genetic diversity among *Berberis* spp. from Karakoram Mountain Ranges using morpho-pathological and floral characters. J. Biodivers. Environ. Sci..

[29] Ahmed M, Anjum MA, Naz RMM, Khan MR, Hussain S (2013). Characterization of indigenous barberry germplasm in Pakistan: variability in morphological characteristics and nutritional composition. Fruits.

[30] Gregory PH (1968). Interpreting plant disease dispersal gradients. Annu. Rev. Phytopathol..

[31] Bueno-Sancho, V. & Saunders, D. G. O. Aeciospore dispersal model, www.aeciospore-dispersal-model.com (2021).

[32] Littlefield, L. J. & Heath, M. C. in *Ultrastructure of Rust Fungi* Ch. 2, 3–91 (Academic Press Inc., 1979).

[33] Yafetto L (2008). The fastest flights in nature: high-speed spore discharge mechanisms among fungi. PLoS ONE.

[34] Cotter, R. U. Factors affecting the development of the aecial stage of *Puccinia graminis*. U. S. Dept. Agr., Tech. Bul. 314 (1932).

[35] UK Met Office. (ed. UK Met Office) (2020).

[36] Prussin AJ, Marr LC, Schmale DG, Stoll R, Ross SD (2015). Experimental validation of a long-distance transport model for plant pathogens: application to *Fusarium graminearum*. Agr. For. Meteorol..

[37] Kuparinen A (2006). Mechanistic models for wind dispersal. Trends Plant Sci..

[38] Dodge BO (1924). Aecidiospore discharge as related to the character of the spore wall. J. Agric. Res..

[39] Holovaty, A. & Kaplan-Moss, J. *The Definitive Guide to Django: Web Development Done Right*. 2nd edn, 536 (Apress, 2009).

[40] Weatherbit. Weather API - Historical Weather API, https://www.weatherbit.io/ (2020).

[41] Bueno-Sancho, V. *Imaging*, https://github.com/vbuens/imaging/ (2020).

[42] Bueno-Sancho, V. *Imaging*, 10.5281/zenodo.5508137 (2021).

[43] Bueno-Sancho, V. aeciospore-dispersal, https://github.com/vbuens/aeciospore-dispersal (2020).

[44] Bueno-Sancho, V. *aeciospore-dispersal: First release of aeciospore dispersal website*, 10.5281/zenodo.5508133 (2021).

