# Supplementary material for: Aeciospore ejection in the rust pathogen Puccinia graminis is driven by moisture ingress
Source: Commun Biol. 2021 Oct 22;4:1216. doi: 10.1038/s42003-021-02747-1 (PMC8536709; doi:10.1038/s42003-021-02747-1)
Supplement: Supplementary file 1 — Supporting Information [file 42003_2021_2747_MOESM1_ESM.pdf]

COMMUNICATIONS BIOLOGY: Supplementary Information

Aeciospore Ejection in the Rust Pathogen *Puccinia graminis* Is Driven by Moisture Ingress

Vanessa Bueno-Sancho, Elizabeth S. Orton, Morgan Gerrity, Clare M. Lewis, Phoebe Davey, Kim Findlay, Elaine Barclay, Phil Robinson, Richard J. Morris, Mark Blyth, Diane G.O. Saunders

The following Supplementary Information is available for this article:

Supplementary Note 1.

Fig. S1. Number of cups per *Pg* aecium and length of aecial cups.

Fig. S2. The interspore gap was generally consistent irrespective of the position of *Pg* aeciospores within aecial cups.

Fig. S3. Contour plots illustrate that predicted aeciospore ejection speeds only considerably increased when altering the mass parameter to represent release of aeciospores in clusters or chains.

Fig. S4. Aeciospore ejection speed achieved after an initial burst of acceleration varies with the power law exponent γ.

Fig. S5. Experimental set-up for high-speed videography.

Fig. S6. The initial ejection speed of *Pg* aeciospores rapidly decreased as drag force was exerted.

Fig. S7. Aeciospores released in clusters travelled longer distances.

Fig. S8. The cumulative number of *Pg* aeciospores released showed a normal distribution for the range of temperatures tested (5–30 ºC).

Table S1. The predicted *Pg* aeciospore release speeds required to achieve the observed distance travelled were higher than those observed using high-speed videography.

Table S2. Number of aeciospores released per *Pg* aecium under different temperatures.

Supplementary Note 1

1. Estimating the pressure force per unit length on the edge of the aeciospore. Generating a substantial force that is exerted on neighboring aeciospores is crucial to the process of aeciospore release, acting to propel aeciospores forward in the direction of the axis of symmetry i.e., the positive *x* direction (Fig. **2b**). We modelled this process using a modified form of the Reynolds lubrication equation[1]:

$$\frac{1}{12\mu}\frac{\partial}{\partial z}\left(H^3\frac{\partial p}{\partial z}\right) = V^{(y)} - \frac{1}{2}V^{(z)}\frac{\partial h}{\partial z}. \tag{1}$$

where $p(z,t)$ is the pressure in the liquid film, μ is the dynamic viscosity of water (8.9 x 10^{-4} Pa s), $V^{(y)}$ and $V^{(z)}$ are the velocity components of the spore in the y and z directions respectively. The edge of the aeciospore of length L is located at $y = H(t)$ (Fig. **2c**), where $H(t)$ is the time-dependent gap. Considering $x = X(t)$, the location of the center of mass of the aeciospore, then $V^{(y)} = dH/dt$ and $V^{(z)} = \left(\frac{dX}{dt}\right)$ cos α and equation (1) can therefore be written as:

$$\frac{1}{12\mu}\frac{\partial}{\partial z}\left(H^3\frac{\partial p}{\partial z}\right) = \frac{dH}{dt} \tag{2}$$

Integrating with respect to *z*, we obtain:

$$\frac{\partial p}{\partial z} = 12\mu\big(z + C_1(t)\big)\frac{1}{H^3}\frac{dH}{dt} \tag{3}$$

for an arbitrary function $C_1(t)$. Integrating again, we get:

$$p = (6\mu\,(z + C_1)^2 + C_2(t))\frac{1}{H^3}\frac{dH}{dt} \tag{4}$$

for arbitrary function $C_2(t)$. We assume that aeciospores are released following a build-up of pressure caused by the swelling of aeciospores that reaches a sufficient level to break the bonds that hold the aeciospore in place. Accordingly, we allow for a pressure differential, p_0, between the base of the aeciospore (or chain of aeciospores) and the top of the cup, and hence demand that $p = p_0$ at $z = 0$ and $p = 0$ at $z = L$. Using these conditions to fix C_1 and C_2 we find:

$$p(z,t) = \left(1 - \frac{z}{L}\right)\left(\mathrm{p}_0 - 6\mu\frac{L^2}{H^3}\frac{dH}{dt}\frac{z}{L}\right) \tag{5}$$

The total hydrodynamic force due to the fluid pressure that acts on the face of the aeciospore is thus given by $\boldsymbol{P} = P\boldsymbol{n}$, where $\boldsymbol{n}$ is the unit vector that is normal to the aeciospore face and pointing away from the liquid film (Fig. **2c**), and

$$\mathrm{P} = \lambda \int_0^L p\ dz = \frac{1}{2}\lambda L p_0 + \mu\lambda \frac{L^3}{H^3}\left(-\frac{dH}{dt}\right), \tag{6}$$

where λ is the length of the aeciospore in the transverse direction. The aeciospore experiences a viscous drag on its face equal to $\boldsymbol{D} = -D\boldsymbol{e}_z$, where $\boldsymbol{e_z}$ is the unit vector in the z direction and

$$\mathrm{D} = \mu\lambda \int_0^{\mathrm{L}} \left(\frac{\partial \mathrm{u}}{\partial \mathrm{y}}\right)_{\mathrm{y=H}} \mathrm{dz}, \tag{7}$$

where $u(y, t)$ is the component of the fluid velocity in the film in the z direction given by

$$\mathrm{u}(y, \mathrm{t}) = \frac{1}{2\mu}\mathrm{y}(\mathrm{y} - \mathrm{H})\frac{\partial \mathrm{p}}{\partial \mathrm{z}} + \mathrm{V}^{(\mathrm{z})}\frac{\mathrm{y}}{\mathrm{H}}. \tag{8}$$

Therefore

$$\mathrm{D} = -\frac{1}{2}\lambda \mathrm{H} \mathrm{p}_0 + \mu\lambda \frac{\mathrm{L}}{\mathrm{H}}\frac{\mathrm{dX}}{\mathrm{dt}}\cos\alpha. \tag{9}$$

Including the pressure force and the viscous drag, the total hydrodynamic force acting on the aeciospore in the x direction, accounting for each of the four faces that oppose aeciospores in adjacent chains, is $\lambda^2 p_0 + 4F$, where the term $\lambda^2 p_0$ arises due to the pressure p_0 acting on the bottom face of the aeciospore, and

$$\mathrm{F} \equiv (\boldsymbol{P} + \boldsymbol{D}) \cdot \boldsymbol{e}_x = P\sin\alpha - D\cos\alpha, \tag{10}$$

where $\boldsymbol{e}_x$ is the unit vector in the x direction. Newton's second law yields the equation of motion of the aeciospore of mass m,

$$\frac{d^2X}{dt^2} + A\frac{dX}{dt} + B = 0, \tag{11}$$

where

$$A = \frac{4\mu\lambda}{m}\frac{L}{H}\cos^2\alpha,$$

$$B = -\frac{4\mu\lambda}{m}\frac{L^3}{H^3}\left(-\frac{dH}{dt}\right)\sin\alpha - \frac{2\lambda}{m}(L\sin\alpha + 2\lambda + H\cos\alpha)p_0. \tag{12}$$

It is convenient to non-dimensionalise equation (11) by writing $X = \chi L$, $H = hL$ and $t = T\tau$ where $T = m/4\mu\lambda$ represents a natural time scale. With $\rho = 882\ kg\ m^{-3}$ for the aeciospore density and $\lambda = 13.9 \times 10^{-6} m$ for the aeciospore size and using $\mu = 8.0 \times 10^{-4}\ Pa\ s$ for the viscosity of water, this gives a time scale of $T = 4.8 \times 10^{-5}$s. Consider first the case of a negligible pressure differential, $p_0 = 0$. Assuming α is small, the non-dimensionalized equation of motion (11) takes the form

$$\frac{d^2\chi}{d\tau^2} = \frac{1}{h}\left[\frac{\alpha}{h^2}\left(-\frac{dh}{d\tau}\right) - \frac{d\chi}{d\tau}\right]. \tag{13}$$

The first term in the square bracket on the right-hand side represents the lubrication force that drives the aeciospore out of the cup as the gap closes $\left(\frac{dh}{d\tau} < 0\right)$, and the second term in the square bracket represents the retarding force due to the viscous drag. We hypothesise that the gap closes according to a power law so that

$$\frac{dh}{d\tau} = -bh^{\gamma} \qquad (14)$$

for some constants b and γ. Then if $\gamma \neq 1$

$$h(\tau) = h_0(1 + \tilde{\kappa}\tau)^{-1/(\gamma-1)}, \qquad \tilde{\kappa} = b(\gamma - 1)h_0^{\gamma-1}, \qquad (15)$$

where $h_0 = h(0)$. If $0 < \gamma < 1$ the interspore gap will close in finite time. If $\gamma > 1$ the gap will close in infinite time. According to (13) if $\gamma > 2$ the speed of the aeciospore will reach a maximum before slowly decreasing to zero. We will assume that $1 < \gamma \leq 2$ so that the interspore gap closes in infinite time and the aeciospore accelerates monotonically in time. In the special case $\gamma = 2$ the aeciospore reaches the terminal velocity

$$v_{\infty} \equiv \left(\frac{4\mu\lambda L}{m}\right) \lim_{\tau\to\infty} \frac{d\chi}{d\tau} = \left(\frac{4\mu\lambda L}{m}\right) \alpha b. \qquad (16)$$

For general γ and α we may calculate the ejection velocity

$$v^* \equiv \left(\frac{4\mu\lambda L}{m}\right) \frac{d\chi}{d\tau}\bigg|_{\chi=\chi^*}, \qquad (17)$$

where $\chi^* = \frac{k\lambda}{L}$. This corresponds to the velocity attained by the aeciospore after it has travelled k aeciospore lengths. By solving equation (11) numerically in Matlab using the inbuilt function *ode45* we calculated the ejection speed v^* over a range of values of α and γ by taking $b = 1$ and using the physical parameter values $\lambda = 13.9 \times 10^{-6} m$, $\mu = 8.0 \times 10^{-4}\, Pa\, s$ (viscosity of water), $\rho = 882\, kg\, m^{-3}$ (spore density) and $H_0 = H(0) = 1.96\ 10^{-6} m$ (initial gap). We assumed that the aeciospore started from rest so that $\chi(0) = \frac{d\chi}{d\tau}(0) = 0$ and calculated values of v^* for a range of (α, γ) values for a single aeciospore with mass $m = \rho\lambda^3$. We then predicted values of v^* for a chain of five aeciospores end-to-end and a $3 \times 3 \times 3$ cubic cluster comprising 27 aeciospores in total (Supplementary Fig. **S3**). The predicted ejection speeds for aeciospores released as chains or clusters were in line with those observed experimentally (Fig. **3a–b**). To demonstrate the robustness of these predictions with respect to changes in the parameters, we assessed the ejection speed whilst varying the initial gap H_0 for the sample power law exponent $\gamma = 1.25$ for three different ejection angles α

(Supplementary Fig. **S3**). In all three cases the predicted ejection speeds were broadly in line with experimental measurements.

Turning now to the effect of a non-zero base pressure, $p_0 \neq 0$, the non-dimensionalised equation (11) becomes (for α small),

$$\frac{d^2\chi}{d\tau^2} = \frac{1}{h}\left[\frac{\alpha}{h^2}\left(-\frac{dh}{d\tau}\right) - \frac{d\chi}{d\tau}\right] + \frac{m}{8\mu^2 L}\left(2 + \frac{L}{\lambda}h\right)p_0. \qquad (18)$$

We then assessed ejection velocities over a range of ejection angles al for a single aeciospore assuming the power law gap-closure relation (15) with $\gamma = 1.25$ (Supplementary Fig. **S3**). As expected, the general trend is that a smaller base pressure p_0 is needed to achieve the same ejection at a larger ejection angle α. At zero ejection angle, $\alpha = 0$, a pressure differential p_0 of $\frac{1}{4}$ of an atmosphere (0.5 Nm^{-2}) is sufficient to eject the aeciospore at speed $v^* = 0.4\ \mathrm{ms}^{-1}$. However, it should be emphasised that at a zero ejection angle, $\alpha = 0$, there is no propulsive lubrication force and the viscous drag will cause the aeciospore to reach a maximum speed prior to entering a deceleration phase. Numerical calculations for $\alpha = 0$ show that the maximum speed is likely to be reached and the deceleration phase to be entered prior to the exit of the aeciospore from the cup (e.g. in less than one aeciospore's length for a single aeciospore) even when p_0 is sufficiently large to achieve an ejection speed at the higher end of the experimentally observed values (i.e. up to about 0.7 $m \cdot s^{-1}$).

A difficulty with the previous calculation is that as the gap squeezes shut the lubrication pressure eventually becomes large enough to drive fluid in the negative *z* direction towards the base of the aecial cup. To address this, we instead assume that the base pressure p_0 adjusts dynamically to prevent backflow. Using the formulae for the fluid velocity and pressure given above, the flux in the gap,

$$\mathrm{q} = \int_0^{\mathrm{H}} \mathrm{u}(\mathrm{y},\mathrm{t})\ \mathrm{dy} = \frac{\mathrm{p_0 H^3}}{12\mu\mathrm{L}} + \frac{1}{2}\left(-\frac{\mathrm{dH}}{\mathrm{dt}}\right)(2\mathrm{z} - \mathrm{L}) + \frac{1}{2}\mathrm{H}\frac{\mathrm{dX}}{\mathrm{dt}}\cos\alpha \qquad (19)$$

Then the required end pressure

$$p_0 = -\frac{6\mu L}{H^3}\left(L\frac{dH}{dt} + H\frac{dX}{dt}\cos\alpha\right). \qquad (20)$$

Nondimensionalizing using the same scales introduced above, the equation of motion is then given by (for $\alpha \ll 1$)

$$\frac{d^2\chi}{d\tau^2} = -\left[\frac{1}{h} + \frac{3}{h^2}\left(\frac{2\lambda}{L} + h + \alpha\right)\right]\frac{d\chi}{d\tau} + \frac{3}{h^3}\left(\frac{2\lambda}{L} + h + \frac{4}{3}\alpha\right)\left(-\frac{dh}{d\tau}\right). \tag{21}$$

Assuming a power law behaviour for the gap closure as above, namely $dh/d\tau = -bh^{\gamma}$, the aeciospore will accelerate very rapidly up to some velocity, which has only a weak dependence on α, before entering a much gentler phase in which velocity varies relatively slowly, before the aeciospore exits the cup (Supplementary Fig. **S4**). The predicted values are broadly in line with the experimental observations over a range of γ values.

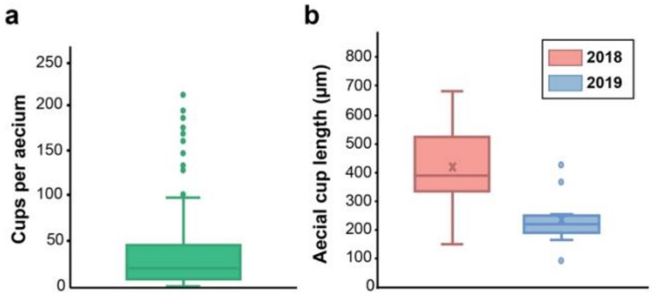

Fig. S1. Number of cups per *Pg* aecium and length of aecial cups. (a) Analysis of 129 *Pg* aecia from three independent sites showed that each aecium on average consisted of 40 individual cups (S.D. ± 48). n = 129. **(b)** Aecial cups varied greatly in length depending on environmental conditions, with more elongated aecial cups observed in 2018 (n = 39) compared to 2019 (n = 12), coinciding with a cooler early spring in the UK. A significant difference was found when comparing aecial cup lengths between 2018 (average 417.9 µm) and 2019 (average 235.1 µm) (p-value < 0.05; *t*-test). Bar represents median value, box signifies the upper (Q3) and lower (Q1) quartiles, whiskers are located at 1.5 the inter-quartile range.

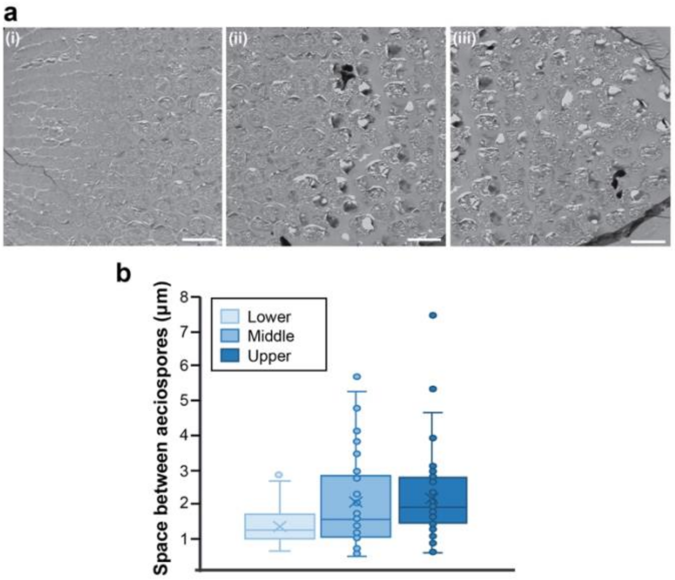

Fig. S2. The interspore gap was generally consistent irrespective of the position of *Pg* aeciospores within aecial cups. (a) Transmission electron micrographs illustrating the space between aeciospores at the (i) lower (ii), middle and (iii) upper position within an aecial cup. **(b)** Measurements of interspore gaps showed no significant difference between positions within the aecium. A total of 29 (lower), 42 (middle) and 67 (upper) measurements were taken of interspore gaps, with an average of 1.4, 2.0 and 2.2 μm respectively. Bar represents median value, box signifies the upper (Q3) and lower (Q1) quartiles, whiskers are located at 1.5 the inter-quartile range.

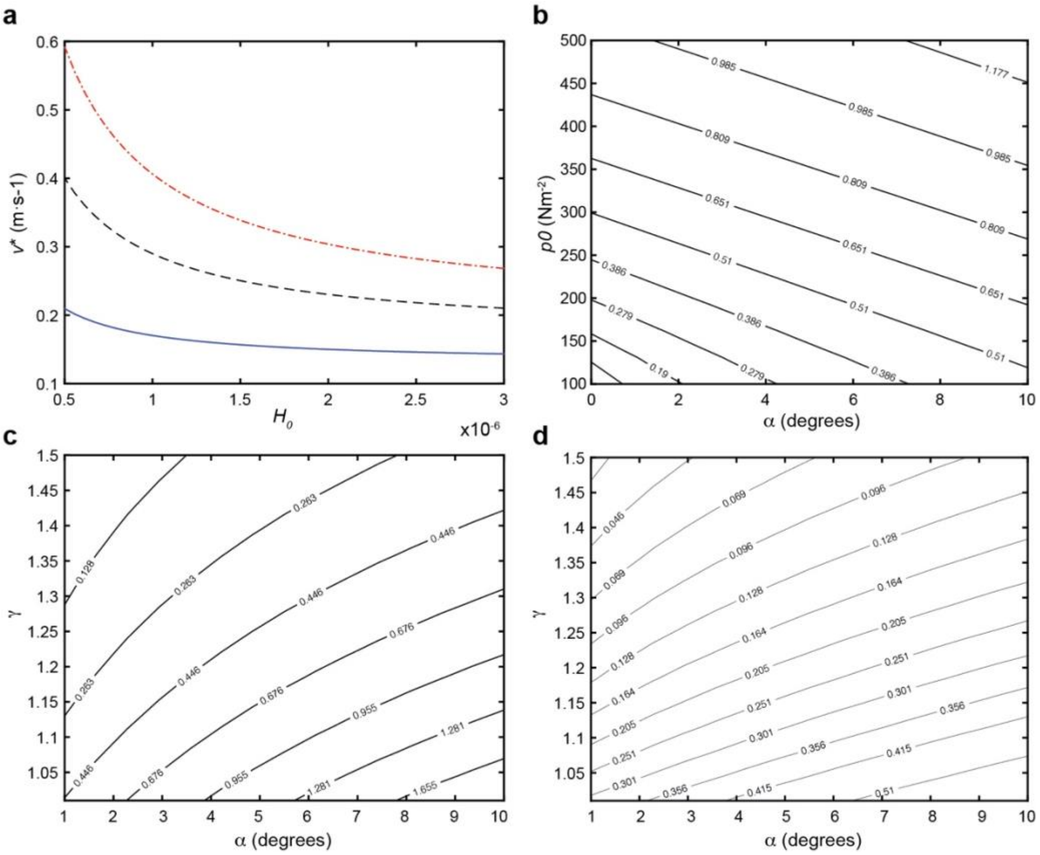

Fig. S3. Contour plots illustrate that predicted aeciospore ejection speeds only considerably increased when altering the mass parameter to represent release of aeciospores in clusters or chains. (a) Predicted ejection velocities v^* (with $k = 1$) in m·s^{-1} for a single aeciospore when $\gamma = 1.25$ for ejection angle $\alpha = 2°$ (blue, solid curve), $\alpha = 5°$ (black, dashed curve) and $\alpha = 8°$ (red dot-dashed curve). **(b)** v^* (with $k = 1$) in m·s^{-1} for a single aeciospore of mass $m = \rho\lambda^3$ for power law exponent $\gamma = 1.25$ over a range of ejection angles α and base pressures p_0. **(c)** v^* (with $k = 5$) in m·s^{-1} for a 5×1 chain of aeciospores of dimension and mass $m = \rho\lambda^2 L$ with $L = 5\lambda$. **(d)** v^* (with $k = 5$) in m·s^{-1} for a $3 \times 3 \times 3$ cubic cluster of 27 aeciospores of dimension 5λ and mass $m = 125\rho\lambda^3$.

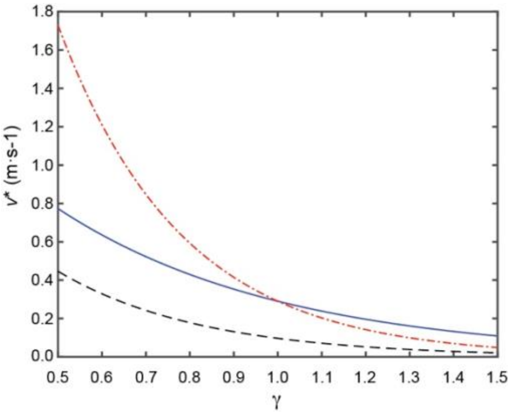

Fig. S4. Aeciospore ejection speed achieved after an initial burst of acceleration varies with the power law exponent γ. Predicted ejection velocities $\boldsymbol{v}^*$ in m·s^{-1} when assuming a power law behavior for the gap closure, namely $\boldsymbol{dh/d\tau = -bh^{\gamma}}$, for a small $\boldsymbol{\alpha}$ and for single aeciospores with $\boldsymbol{b}$ = 1 and the base pressure $\boldsymbol{p_0}$ is chosen to ensure zero flux at the base of the aeciospores. Single aeciospore (blue solid line), 3 × 3 × 3 cubic cluster of 27 aeciospores (black dashed line), 5 × 1 chain of aeciospores (red dot-dashed line).

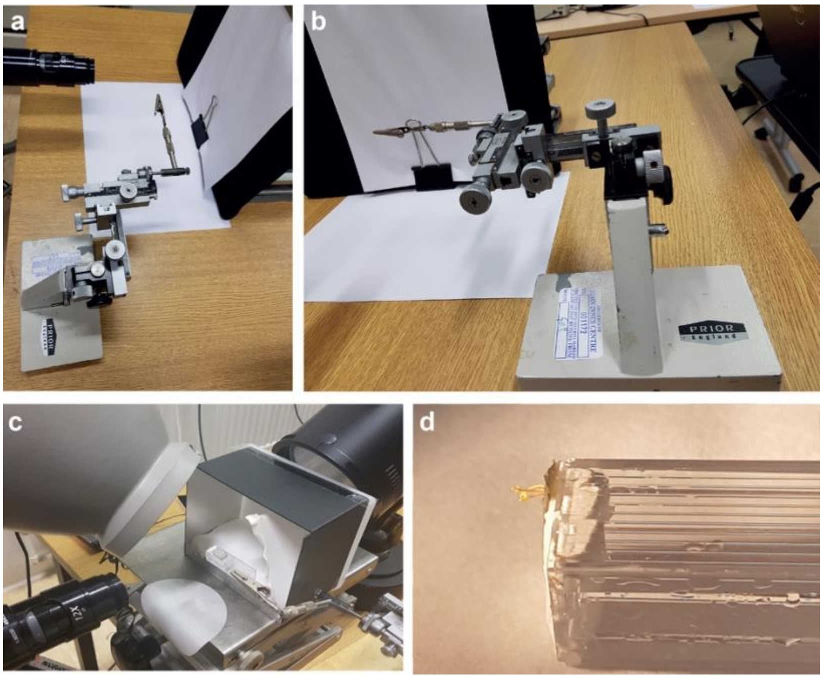

Fig. S5. Experimental set-up for high-speed videography. **(a)** The Photron Fastcam SA-X2 mono high-speed camera with a Navitar 12x zoom lens was positioned facing a white background. **(b)** A clamp was used for fine control of aecia positioning. **(c)** Two LED beam lights were positioned nearby to illuminate the sample and thereby enable high-speed videography. **(d)** Leaf fragments of *B. vulgaris* that displayed yellow, tube-like aecial structures were identified, cut and attached to a cuvette using Vaseline®. The cuvette was then held by the clamp and positioned to enable visualization.

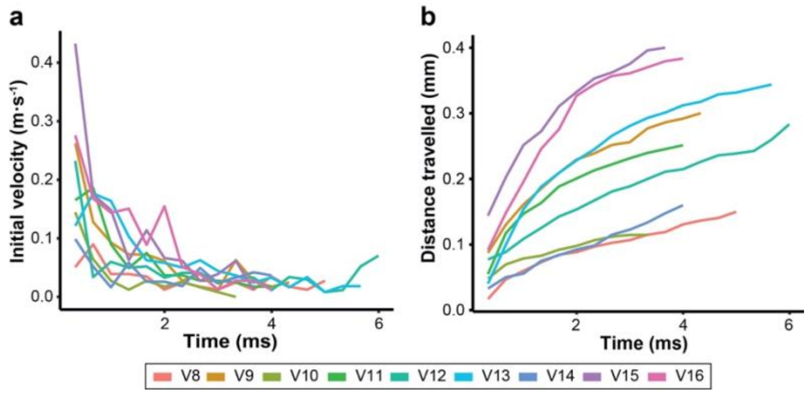

Fig. S6. The initial ejection speed of *Pg* aeciospores rapidly decreased as drag force was exerted. (a–b) After ejection, aeciospore speed rapidly decreased over time. Aeciospore speed of discharge was measured frame by frame until aeciospores began to decelerate as drag force was exerted. V8–16 represent independent aeciospore release events.

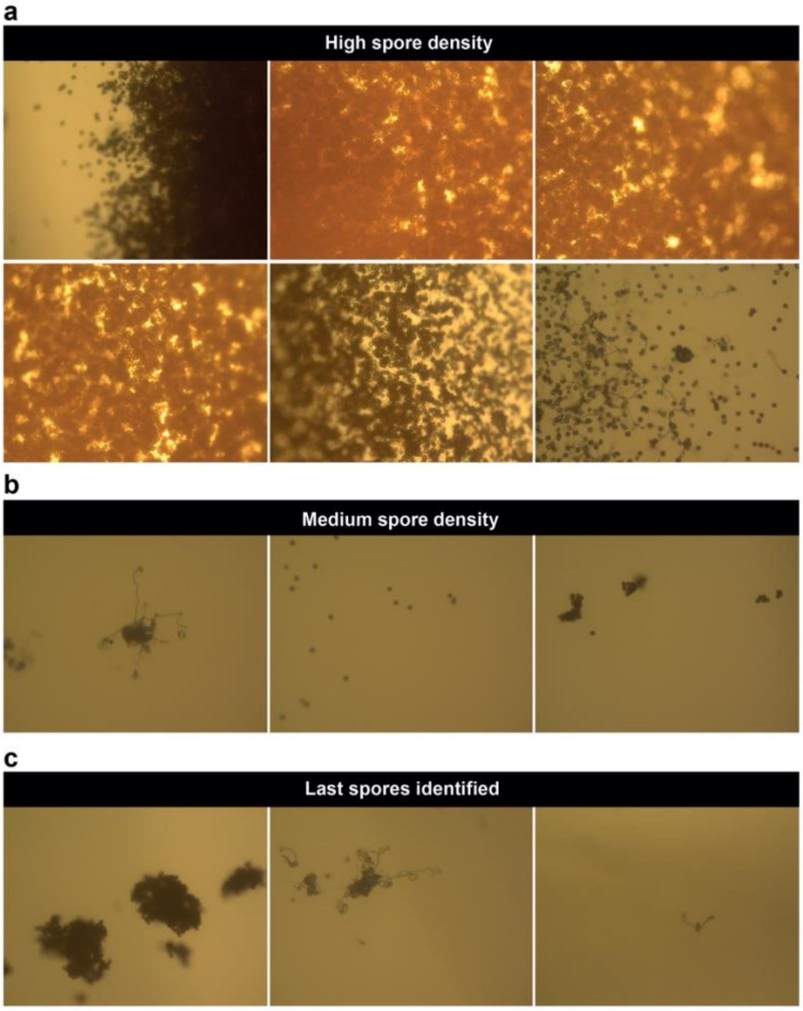

Fig. S7. Aeciospores released in clusters travelled longer distances. *Pg* aecial cups were incubated for 18 hours in darkness at 18 ºC and slides positioned adjacent to capture released aeciospores. Aeciospore release speed was determined using maximum distances from **(a)** high spore density (> 100 spores/0.2 mm^2), **(b)** medium spore density (10–50 spores/0.2 mm^2), and **(c)** final maximum distance (last spores identified). Aeciospores identified at greater distances were consistently found in clusters; this would increase the mass-air drag (m/ζ ratio), thereby facilitating greater distances of travel.

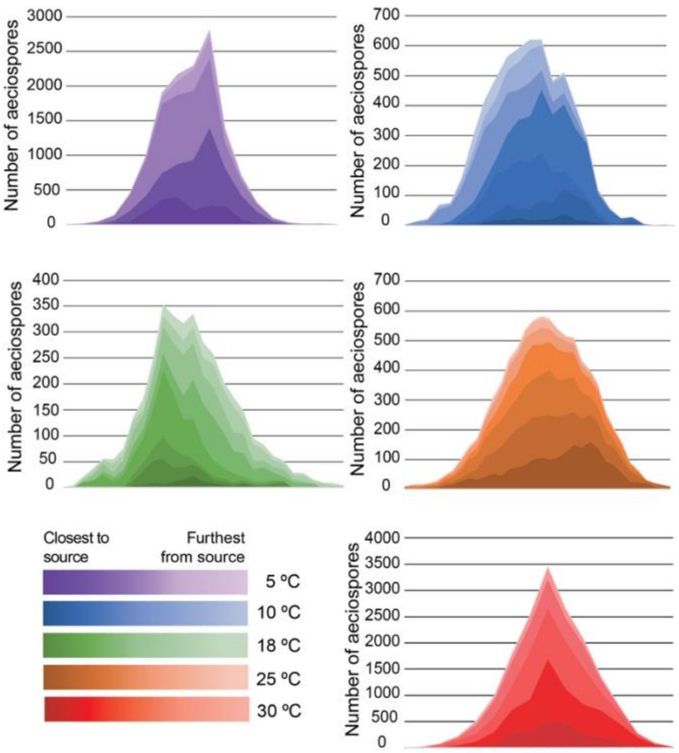

Fig. S8. The cumulative number of *Pg* aeciospores released showed a normal distribution for the range of temperatures tested (5–30 ºC). Aecial cups were incubated for 18 hours in darkness at 5 ºC, 10 ºC, 18 ºC, 25 ºC and 30 ºC and slides positioned adjacent to capture released aeciospores. Three replicates for each temperature were undertaken and aeciospores counted in rows from the source, with counts of aeciospores per row then stacked from closest to furthest from the source.

Table S1. The predicted *Pg* aeciospore release speeds required to achieve the observed distance travelled were higher than those observed using high-speed videography. Initial *Pg* aeciospore ejection speed was predicted from the observed maximum distance aeciospores travelled.

**Spore density**	**Distance (cm)**	**Predicted speed ($m \cdot s^{-1}$)**
Max. high spore density	1.2	11.01
Max. medium spore density	2	18.35
Max. maximum distance	4.8	44.04

Table S2. Number of aeciospores released per *Pg* aecium under different temperatures. Three independent aecia were incubated at each temperature for 18 hours. The size of the aecium was evaluated ("area") and used to estimate the potential number of aeciospores that could be released. The total number of *Pg* aeciospores actually released was then determined. The ratio between the number of aeciospores predicted to be released and the true value was assessed.

**Temp. ºC**	**Replicate**	**Area (mm^2)**	**Estimated aeciospores**	**Aeciospores released**	**Ratio released vs. estimated (%)**
5	1	30	1768505.7	17882	1.01
5	2	12	707402.28	13458	1.9
5	3	6	353701.14	12088	3.42
10	1	20	1179003.8	10443	0.89
10	2	20	1179003.8	55226	4.68
10	3	9	530551.71	67523	12.73
18	1	6	353701.14	7404	2.09
18	2	9	530551.71	3594	0.68
18	3	25	1473754.75	16309	1.11
25	1	6	353701.14	8319	2.35
25	2	16	943203.04	10613	1.13
25	3	12	707402.28	6407	0.91
30	1	9	530551.71	14133	2.66
30	2	6	353701.14	16956	4.79
30	3	4	235800.76	4988	2.12
				Average:	2.83

SUPPLEMENTARY REFERENCES

1. Batchelor, G. K. *An introduction to fluid dynamics*. (Cambridge University Press, 1967).
