# Supplementary material for: Aeciospore ejection in the rust pathogen Puccinia graminis is driven by moisture ingress
Source: Commun Biol. 2021 Oct 22;4:1216. doi: 10.1038/s42003-021-02747-1 (PMC8536709; doi:10.1038/s42003-021-02747-1)
Supplement: Supplementary file 2 — Description of Additional Supplementary Files [file 42003_2021_2747_MOESM2_ESM.pdf]

Description of Additional Supplementary Files

File name: Supplementary Movie S1

Description: Video of *Pg* aeciospore expansion following addition of water. Time duration 7 s and 20 frames per second.

File name: Supplementary Movie S2

Description: High-speed video of *Pg* aeciospore ejection. Time duration 11.2 ms and 20 frames per second.

File name: Supplementary Movie S3

Description: High-speed video of *Pg* aeciospore ejection. Time duration 78.66 ms and 20 frames

per second.

File name: Supplementary Data 1

Description: Details of statistical analyses performed.

File name: Supplementary Data 2

Description: All raw measurements to support the analyses presented.
